# Supplementary material for: Effects of a time-use intervention in isolated patients with coronavirus disease 2019: A randomized controlled study
Source: PLoS One. 2023 Jun 23;18(6):e0287118. doi: 10.1371/journal.pone.0287118 (PMC10289446; doi:10.1371/journal.pone.0287118)

Model Information	
**Data Set**	WORK.JUNG_DATA2
**Dependent Variable**	KLBI_sum
**Covariance Structure**	Unstructured
**Subject Effect**	Subjectid
**Estimation Method**	REML
**Residual Variance Method**	None
**Fixed Effects SE Method**	Model-Based
**Degrees of Freedom Method**	Between-Within

Class Level Information		
**Class**	**Levels**	**Values**
**Subjectid**	41	1 2 4 5 6 7 8 10 11 12 13 14 15 16 17 18 19 22 23 24 25 26 27 28 29 30 31 32 33 34 36 37 39 40 41 42 43 44 45 46 50
**group**	2	1 2
**Time**	2	1 2

Dimensions	
**Covariance Parameters**	3
**Columns in X**	9
**Columns in Z**	0
**Subjects**	41
**Max Obs per Subject**	2

Number of Observations	
**Number of Observations Read**	82
**Number of Observations Used**	82
**Number of Observations Not Used**	0

Iteration History			
**Iteration**	**Evaluations**	**-2 Res Log Like**	**Criterion**
**0**	1	79.09536513	
**1**	1	76.09897334	0.00000000

Convergence criteria met.

Covariance Parameter Estimates		
**Cov Parm**	**Subject**	**Estimate**
**UN(1,1)**	Subjectid	0.1661
**UN(2,1)**	Subjectid	0.02533
**UN(2,2)**	Subjectid	0.1105

Fit Statistics	
**-2 Res Log Likelihood**	76.1
**AIC (Smaller is Better)**	82.1
**AICC (Smaller is Better)**	82.4
**BIC (Smaller is Better)**	87.2

Null Model Likelihood Ratio Test		
**DF**	**Chi-Square**	**Pr > ChiSq**
2	3.00	0.2235

Type 3 Tests of Fixed Effects				
**Effect**	**Num DF**	**Den DF**	**F Value**	**Pr > F**
**group**	1	39	1.77	0.1917
**Time**	1	39	5.77	0.0212
**group*Time**	1	39	14.12	0.0006

Class Level Information		
**Class**	**Levels**	**Values**
**group**	2	1 2
**Time**	2	1 2

**Number of Observations Read**	82
**Number of Observations Used**	82

Dependent Variable: KLBI_sum

Source	DF	Sum of Squares	Mean Square	F Value	Pr > F
**Model**	3	2.39727190	0.79909063	5.78	0.0013
**Error**	78	10.78459761	0.13826407		
**Corrected Total**	81	13.18186951			

Dependent Variable: KLBI_sum

R-Square	Coeff Var	Root MSE	KLBI_sum Mean
0.181861	18.13308	0.371839	2.050610

Overall Noncentrality	
Min Var Unbiased Estimate	13.894
Low MSE Estimate	13.528
95% Confidence Limits	(2.8996,36.26)

Proportion of Variation Accounted for	
Eta-Square	0.18
Omega-Square	0.15
95% Confidence Limits	(0.03,0.31)

						Noncentrality Parameter			
Source	DF	Type I SS	Mean Square	F Value	Pr > F	Min Var Unbiased Estimate	Low MSE Estimate	95% Confidence Limits	
group	1	0.28883913	0.28883913	2.09	0.1524	1.04	1.01	0.00	11.7
Time	1	0.51365976	0.51365976	3.72	0.0576	2.62	2.55	0.00	15.2
group*Time	1	1.59477302	1.59477302	11.53	0.0011	10.24	9.97	1.84	29.3

	Total Variation Accounted For				Partial Variation Accounted For			
Source	Semipartial Eta-Square	Semipartial Omega-Square	Conservative 95% Confidence Limits		Partial Eta-Square	Partial Omega-Square	95% Confidence Limits	
group	0.0219	0.0113	0.0000	0.1176	0.0261	0.0131	0.0000	0.1244
Time	0.0390	0.0282	0.0000	0.1481	0.0455	0.0320	0.0000	0.1568
group*Time	0.1210	0.1093	0.0197	0.2576	0.1288	0.1138	0.0219	0.2635

Dependent Variable: KLBI_sum

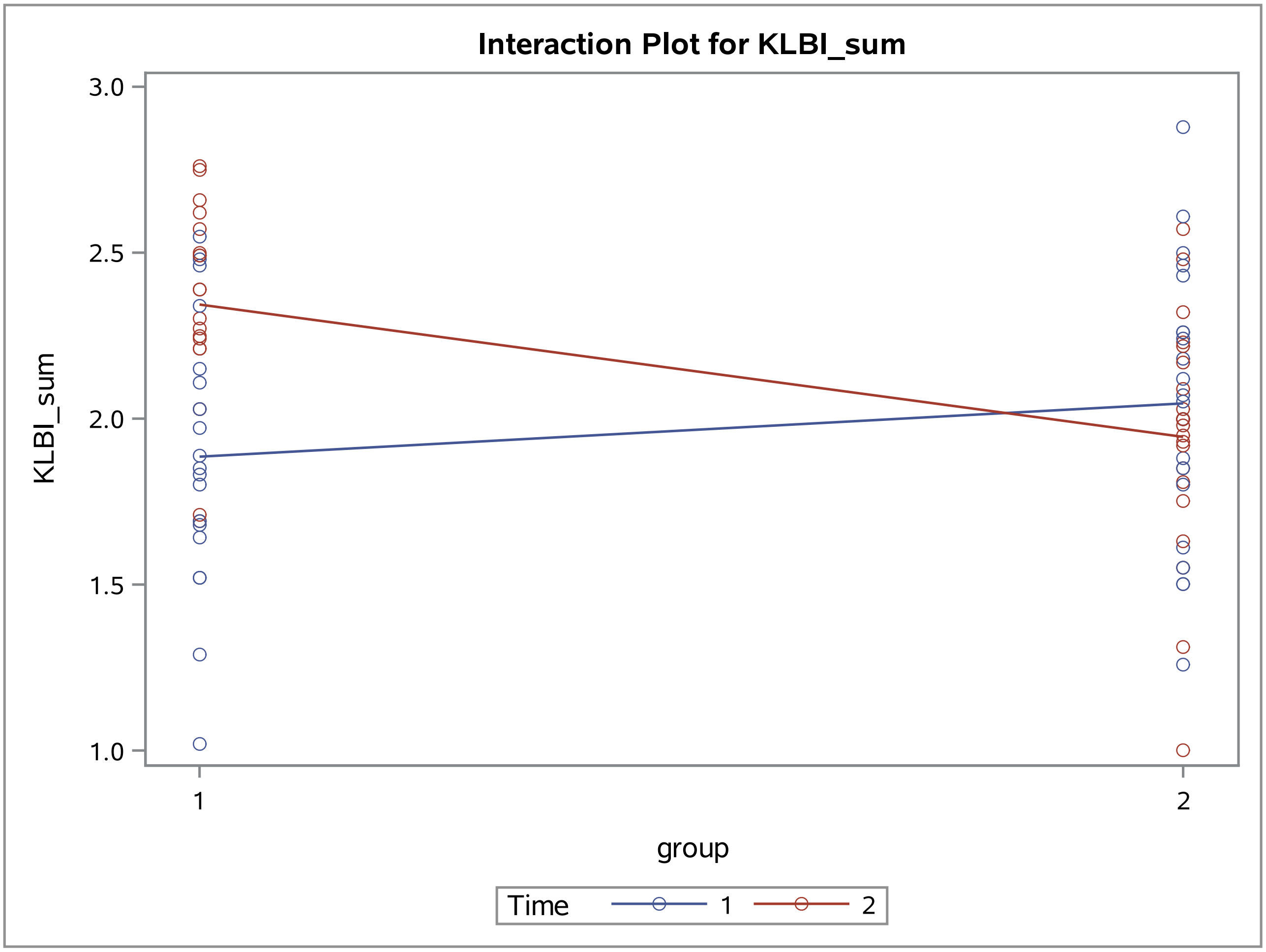

Model Information	
**Data Set**	WORK.JUNG_DATA2
**Dependent Variable**	KLBI_H
**Covariance Structure**	Unstructured
**Subject Effect**	Subjectid
**Estimation Method**	REML
**Residual Variance Method**	None
**Fixed Effects SE Method**	Model-Based
**Degrees of Freedom Method**	Between-Within

Class Level Information

Class	Levels	Values
**Subjectid**	41	1 2 4 5 6 7 8 10 11 12 13 14 15 16 17 18 19 22 23 24 25 26 27 28 29 30 31 32 33 34 36 37 39 40 41 42 43 44 45 46 50
**group**	2	1 2
**Time**	2	1 2

Dimensions	
Covariance Parameters	3
Columns in X	9
Columns in Z	0
Subjects	41
Max Obs per Subject	2

Number of Observations	
Number of Observations Read	82
Number of Observations Used	82
Number of Observations Not Used	0

Iteration History			
Iteration	Evaluations	-2 Res Log Like	Criterion
0	1	90.89644286	
1	1	89.98068163	0.00000000

Convergence criteria met.

Covariance Parameter Estimates		
Cov Parm	Subject	Estimate
UN(1,1)	Subjectid	0.1836
UN(2,1)	Subjectid	0.008989
UN(2,2)	Subjectid	0.1381

Fit Statistics	
-2 Res Log Likelihood	90.0
AIC (Smaller is Better)	96.0
AICC (Smaller is Better)	96.3
BIC (Smaller is Better)	101.1

Null Model Likelihood Ratio Test		
DF	Chi-Square	Pr > ChiSq
2	0.92	0.6326

Type 3 Tests of Fixed Effects				
Effect	Num DF	Den DF	F Value	Pr > F
group	1	39	5.62	0.0227
Time	1	39	3.20	0.0815
group*Time	1	39	10.96	0.0020

Class Level Information		
Class	Levels	Values
group	2	1 2
Time	2	1 2

Number of Observations Read	82
Number of Observations Used	82

Dependent Variable: KLBI_H

Source	DF	Sum of Squares	Mean Square	F Value	Pr > F
Model	3	2.98429244	0.99476415	6.18	0.0008
Error	78	12.54616244	0.16084824		
Corrected Total	81	15.53045488			

R-Square	Coeff Var	Root MSE	KLBI_H Mean
0.192157	17.04599	0.401059	2.352805

Overall Noncentrality	
Min Var Unbiased Estimate	15.078
Low MSE Estimate	14.681
95% Confidence Limits	(3.4618,38.166)

Proportion of Variation Accounted for	
Eta-Square	0.19
Omega-Square	0.16
95% Confidence Limits	(0.04,0.32)

Dependent Variable: KLBI_H

Source	DF	Type I SS	Mean Square	F Value	Pr > F	Noncentrality Parameter			
						Min Var Unbiased Estimate	Low MSE Estimate	95% Confidence Limits	
group	1	0.95527198	0.95527198	5.94	0.0171	4.79	4.66	0.144	19.6
Time	1	0.36488902	0.36488902	2.27	0.1361	1.21	1.18	0.000	12.1
group*Time	1	1.66413143	1.66413143	10.35	0.0019	9.08	8.84	1.399	27.4

Source	Total Variation Accounted For				Partial Variation Accounted For			
	Semipartial Eta-Square	Semipartial Omega-Square	Conservative 95% Confidence Limits		Partial Eta-Square	Partial Omega-Square	95% Confidence Limits	
group	0.0615	0.0506	0.0001	0.1822	0.0708	0.0568	0.0018	0.1928
Time	0.0235	0.0130	0.0000	0.1207	0.0283	0.0152	0.0000	0.1284
group*Time	0.1072	0.0958	0.0138	0.2413	0.1171	0.1023	0.0168	0.2502

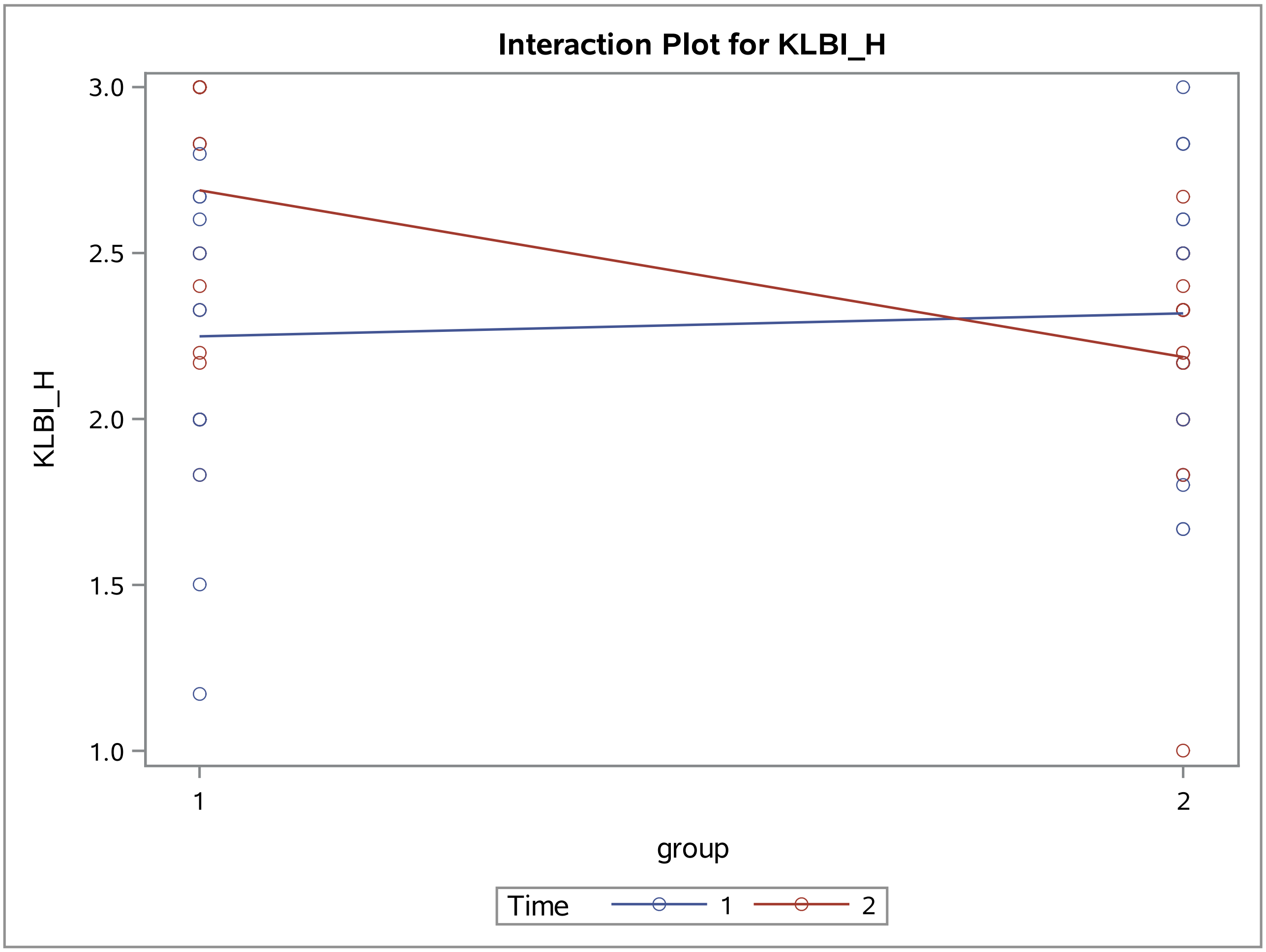

Model Information	
Data Set	WORK.JUNG_DATA2
Dependent Variable	KLBI_R
Covariance Structure	Unstructured
Subject Effect	Subjectid

Model Information	
**Estimation Method**	REML
**Residual Variance Method**	None
**Fixed Effects SE Method**	Model-Based
**Degrees of Freedom Method**	Between-Within

Class Level Information		
**Class**	**Levels**	**Values**
**Subjectid**	41	1 2 4 5 6 7 8 10 11 12 13 14 15 16 17 18 19 22 23 24 25 26 27 28 29 30 31 32 33 34 36 37 39 40 41 42 43 44 45 46 50
**group**	2	1 2
**Time**	2	1 2

Dimensions	
**Covariance Parameters**	3
**Columns in X**	9
**Columns in Z**	0
**Subjects**	41
**Max Obs per Subject**	2

Number of Observations	
**Number of Observations Read**	82
**Number of Observations Used**	81
**Number of Observations Not Used**	1

Iteration History			
**Iteration**	**Evaluations**	**-2 Res Log Like**	**Criterion**
**0**	1	148.74703026	
**1**	2	132.44675803	0.00001024
**2**	1	132.44671150	0.00000000

Convergence criteria met.

Covariance Parameter Estimates		
**Cov Parm**	**Subject**	**Estimate**
**UN(1,1)**	Subjectid	0.4712
**UN(2,1)**	Subjectid	0.1689
**UN(2,2)**	Subjectid	0.2275

Fit Statistics	
-2 Res Log Likelihood	132.4
AIC (Smaller is Better)	138.4
AICC (Smaller is Better)	138.8
BIC (Smaller is Better)	143.6

Null Model Likelihood Ratio Test		
DF	Chi-Square	Pr > ChiSq
2	16.30	0.0003

Type 3 Tests of Fixed Effects				
Effect	Num DF	Den DF	F Value	Pr > F
group	1	39	1.12	0.2959
Time	1	39	1.69	0.2006
group*Time	1	39	3.77	0.0593

Class Level Information		
Class	Levels	Values
group	2	1 2
Time	2	1 2

Number of Observations Read	82
Number of Observations Used	81

Dependent Variable: KLBI_R

Source	DF	Sum of Squares	Mean Square	F Value	Pr > F
Model	3	1.58847968	0.52949323	1.53	0.2130
Error	77	26.61690057	0.34567403		
Corrected Total	80	28.20538025			

R-Square	Coeff Var	Root MSE	KLBI_R Mean
0.056318	30.95228	0.587941	1.899506

Overall Noncentrality	
Min Var Unbiased Estimate	1.476
Low MSE Estimate	1.4366
95% Confidence Limits	(0,14.242)

Dependent Variable: KLBI_R

Proportion of Variation Accounted for	
**Eta-Square**	0.06
**Omega-Square**	0.02
**95% Confidence Limits**	(0.00,0.15)

						Noncentrality Parameter			
Source	DF	Type I SS	Mean Square	F Value	Pr > F	Min Var Unbiased Estimate	Low MSE Estimate	95% Confidence Limits	
**group**	1	0.67086813	0.67086813	1.94	0.1676	0.8903	0.8666	0	11.30
**Time**	1	0.31944813	0.31944813	0.92	0.3394	-0.0999	-0.0972	0	8.54
**group*Time**	1	0.59816342	0.59816342	1.73	0.1923	0.6855	0.6672	0	10.77

	Total Variation Accounted For				Partial Variation Accounted For			
Source	Semipartial Eta-Square	Semipartial Omega-Square	Conservative 95% Confidence Limits		Partial Eta-Square	Partial Omega-Square	95% Confidence Limits	
**group**	0.0238	0.0114	0.0000	0.1220	0.0246	0.0115	0.0000	0.1224
**Time**	0.0113	-0.0009	0.0000	0.0948	0.0119	-0.0009	0.0000	0.0954
**group*Time**	0.0212	0.0088	0.0000	0.1169	0.0220	0.0089	0.0000	0.1174

Dependent Variable: KLBI_R

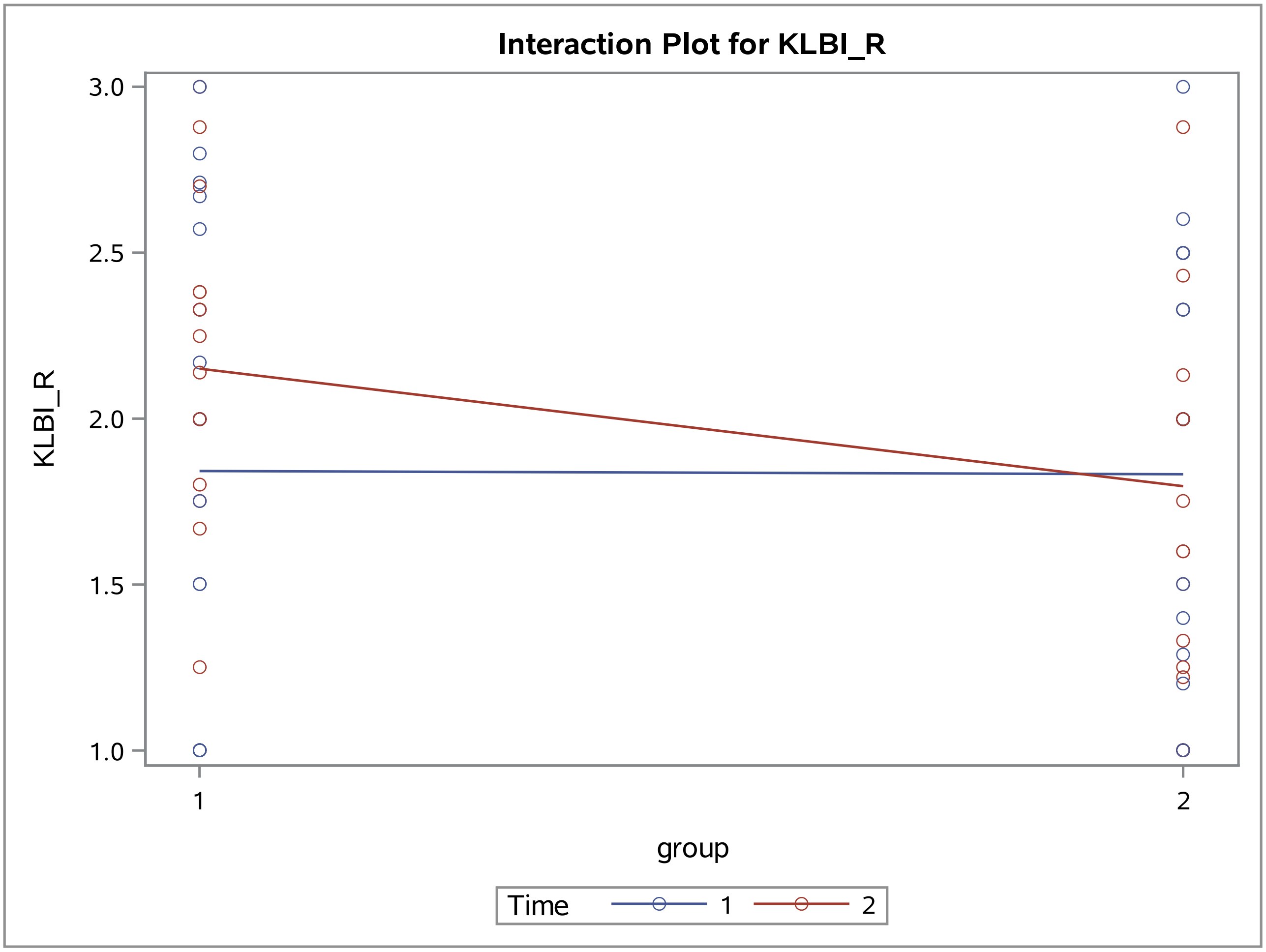

Model Information	
**Data Set**	WORK.JUNG_DATA2
**Dependent Variable**	KLBI_C
**Covariance Structure**	Unstructured
**Subject Effect**	Subjectid
**Estimation Method**	REML
**Residual Variance Method**	None
**Fixed Effects SE Method**	Model-Based
**Degrees of Freedom Method**	Between-Within

Class Level Information

Class	Levels	Values
**Subjectid**	41	1 2 4 5 6 7 8 10 11 12 13 14 15 16 17 18 19 22 23 24 25 26 27 28 29 30 31 32 33 34 36 37 39 40 41 42 43 44 45 46 50
**group**	2	1 2
**Time**	2	1 2

Dimensions	
**Covariance Parameters**	3
**Columns in X**	9
**Columns in Z**	0
**Subjects**	41
**Max Obs per Subject**	2

Number of Observations	
**Number of Observations Read**	82
**Number of Observations Used**	82
**Number of Observations Not Used**	0

Iteration History			
**Iteration**	**Evaluations**	**-2 Res Log Like**	**Criterion**
**0**	1	100.15752861	
**1**	1	96.80763059	0.00000000

Convergence criteria met.

Covariance Parameter Estimates		
**Cov Parm**	**Subject**	**Estimate**
**UN(1,1)**	Subjectid	0.2211
**UN(2,1)**	Subjectid	0.03323
**UN(2,2)**	Subjectid	0.1412

Fit Statistics	
**-2 Res Log Likelihood**	96.8
**AIC (Smaller is Better)**	102.8
**AICC (Smaller is Better)**	103.1
**BIC (Smaller is Better)**	107.9

Null Model Likelihood Ratio Test		
**DF**	**Chi-Square**	**Pr > ChiSq**
2	3.35	0.1873

Type 3 Tests of Fixed Effects				
Effect	Num DF	Den DF	F Value	Pr > F
group	1	39	1.54	0.2216
Time	1	39	5.92	0.0196
group*Time	1	39	12.90	0.0009

Class Level Information		
Class	Levels	Values
group	2	1 2
Time	2	1 2

Number of Observations Read	82
Number of Observations Used	82

Dependent Variable: KLBI_C

Source	DF	Sum of Squares	Mean Square	F Value	Pr > F
Model	3	2.93938817	0.97979606	5.41	0.0020
Error	78	14.12783134	0.18112604		
Corrected Total	81	17.06721951			

R-Square	Coeff Var	Root MSE	KLBI_C Mean
0.172224	21.87433	0.425589	1.945610

Overall Noncentrality	
Min Var Unbiased Estimate	12.812
Low MSE Estimate	12.475
95% Confidence Limits	(2.4034,34.503)

Proportion of Variation Accounted for	
Eta-Square	0.17
Omega-Square	0.14
95% Confidence Limits	(0.03,0.30)

Dependent Variable: KLBI_C

Source	DF	Type I SS	Mean Square	F Value	Pr > F	Noncentrality Parameter			
						Min Var Unbiased Estimate	Low MSE Estimate	95% Confidence Limits	
group	1	0.33072298	0.33072298	1.83	0.1805	0.779	0.759	0.00	11.0
Time	1	0.70068780	0.70068780	3.87	0.0528	2.769	2.696	0.00	15.6
group*Time	1	1.90797739	1.90797739	10.53	0.0017	9.264	9.020	1.47	27.7

Source	Total Variation Accounted For				Partial Variation Accounted For			
	Semipartial Eta-Square	Semipartial Omega-Square	Conservative 95% Confidence Limits		Partial Eta-Square	Partial Omega-Square	95% Confidence Limits	
group	0.0194	0.0087	0.0000	0.1124	0.0229	0.0100	0.0000	0.1184
Time	0.0411	0.0301	0.0000	0.1515	0.0473	0.0338	0.0000	0.1595
group*Time	0.1118	0.1001	0.0157	0.2469	0.1190	0.1042	0.0176	0.2523

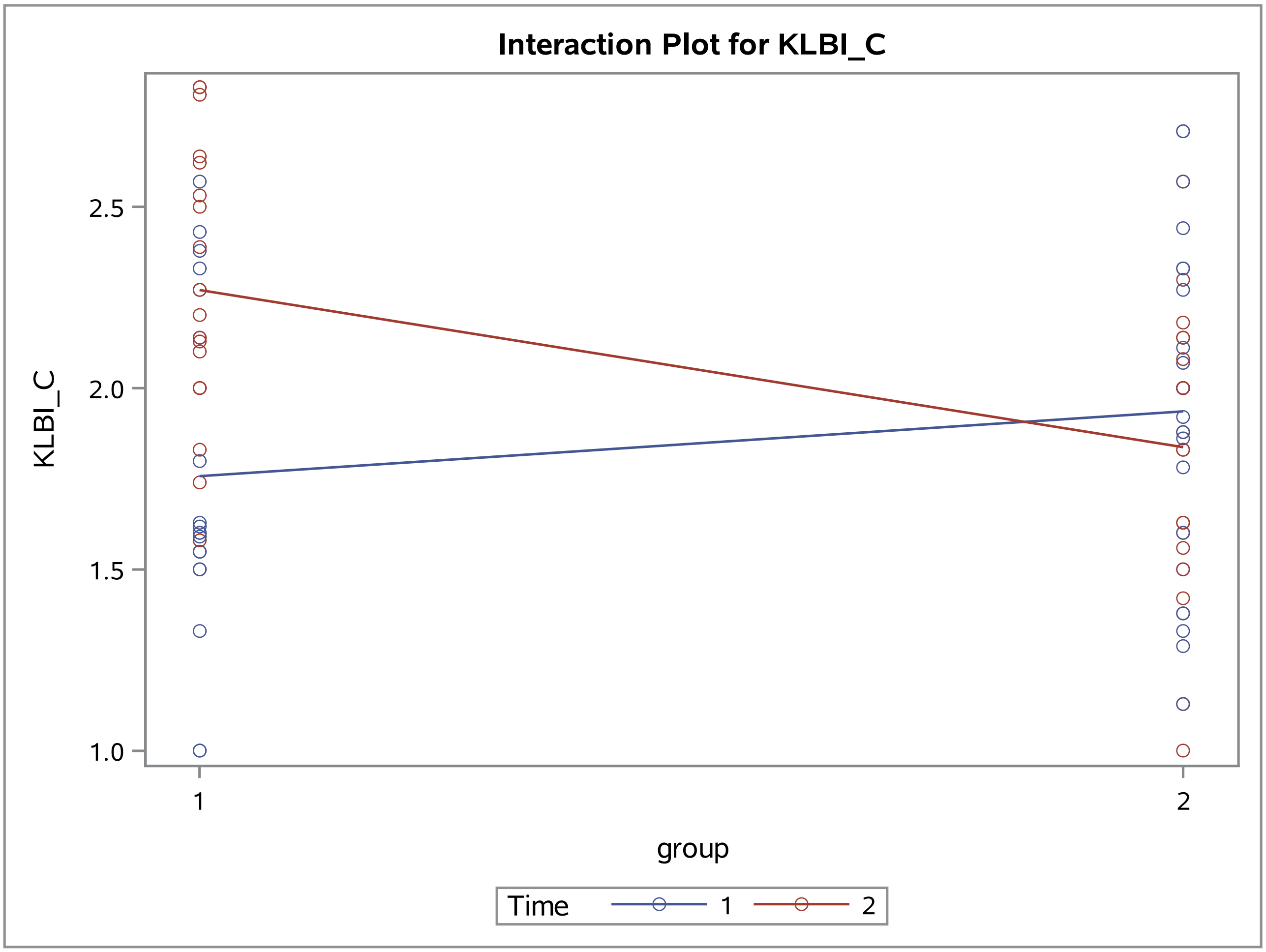

Model Information	
Data Set	WORK.JUNG_DATA2
Dependent Variable	KLBI_I
Covariance Structure	Unstructured
Subject Effect	Subjectid

Model Information	
**Estimation Method**	REML
**Residual Variance Method**	None
**Fixed Effects SE Method**	Model-Based
**Degrees of Freedom Method**	Between-Within

Class Level Information		
**Class**	**Levels**	**Values**
**Subjectid**	41	1 2 4 5 6 7 8 10 11 12 13 14 15 16 17 18 19 22 23 24 25 26 27 28 29 30 31 32 33 34 36 37 39 40 41 42 43 44 45 46 50
**group**	2	1 2
**Time**	2	1 2

Dimensions	
**Covariance Parameters**	3
**Columns in X**	9
**Columns in Z**	0
**Subjects**	41
**Max Obs per Subject**	2

Number of Observations	
**Number of Observations Read**	82
**Number of Observations Used**	82
**Number of Observations Not Used**	0

Iteration History			
**Iteration**	**Evaluations**	**-2 Res Log Like**	**Criterion**
**0**	1	105.46159833	
**1**	1	104.04118038	0.00000000

Convergence criteria met.

Covariance Parameter Estimates		
**Cov Parm**	**Subject**	**Estimate**
**UN(1,1)**	Subjectid	0.2236
**UN(2,1)**	Subjectid	0.02142
**UN(2,2)**	Subjectid	0.1641

Fit Statistics	
-2 Res Log Likelihood	104.0
AIC (Smaller is Better)	110.0
AICC (Smaller is Better)	110.4
BIC (Smaller is Better)	115.2

Null Model Likelihood Ratio Test		
DF	Chi-Square	Pr > ChiSq
2	1.42	0.4915

Type 3 Tests of Fixed Effects				
Effect	Num DF	Den DF	F Value	Pr > F
group	1	39	0.54	0.4678
Time	1	39	5.33	0.0263
group*Time	1	39	12.29	0.0012

Class Level Information		
Class	Levels	Values
group	2	1 2
Time	2	1 2

Number of Observations Read	82
Number of Observations Used	82

Dependent Variable: KLBI_I

Source	DF	Sum of Squares	Mean Square	F Value	Pr > F
Model	3	2.96528496	0.98842832	5.10	0.0028
Error	78	15.12195407	0.19387121		
Corrected Total	81	18.08723902			

R-Square	Coeff Var	Root MSE	KLBI_I Mean
0.163943	21.11419	0.440308	2.085366

Overall Noncentrality	
Min Var Unbiased Estimate	11.903
Low MSE Estimate	11.59
95% Confidence Limits	(2.0006,33.012)

Dependent Variable: KLBI_I

Proportion of Variation Accounted for	
**Eta-Square**	0.16
**Omega-Square**	0.13
**95% Confidence Limits**	(0.02,0.29)

Source	DF	Type I SS	Mean Square	F Value	Pr > F	Noncentrality Parameter			
						Min Var Unbiased Estimate	Low MSE Estimate	95% Confidence Limits	
**group**	1	0.11572180	0.11572180	0.60	0.4421	-0.418	-0.407	0.00	7.45
**Time**	1	0.73058049	0.73058049	3.77	0.0558	2.672	2.601	0.00	15.36
**group*Time**	1	2.11898267	2.11898267	10.93	0.0014	9.650	9.396	1.61	28.34

Source	Total Variation Accounted For				Partial Variation Accounted For			
	Semipartial Eta-Square	Semipartial Omega-Square	Conservative 95% Confidence Limits		Partial Eta-Square	Partial Omega-Square	95% Confidence Limits	
**group**	0.0064	-0.0043	0.0000	0.0801	0.0076	-0.0049	0.0000	0.0833
**Time**	0.0404	0.0294	0.0000	0.1504	0.0461	0.0327	0.0000	0.1577
**group*Time**	0.1172	0.1053	0.0180	0.2532	0.1229	0.1080	0.0193	0.2568

Dependent Variable: KLBI_I

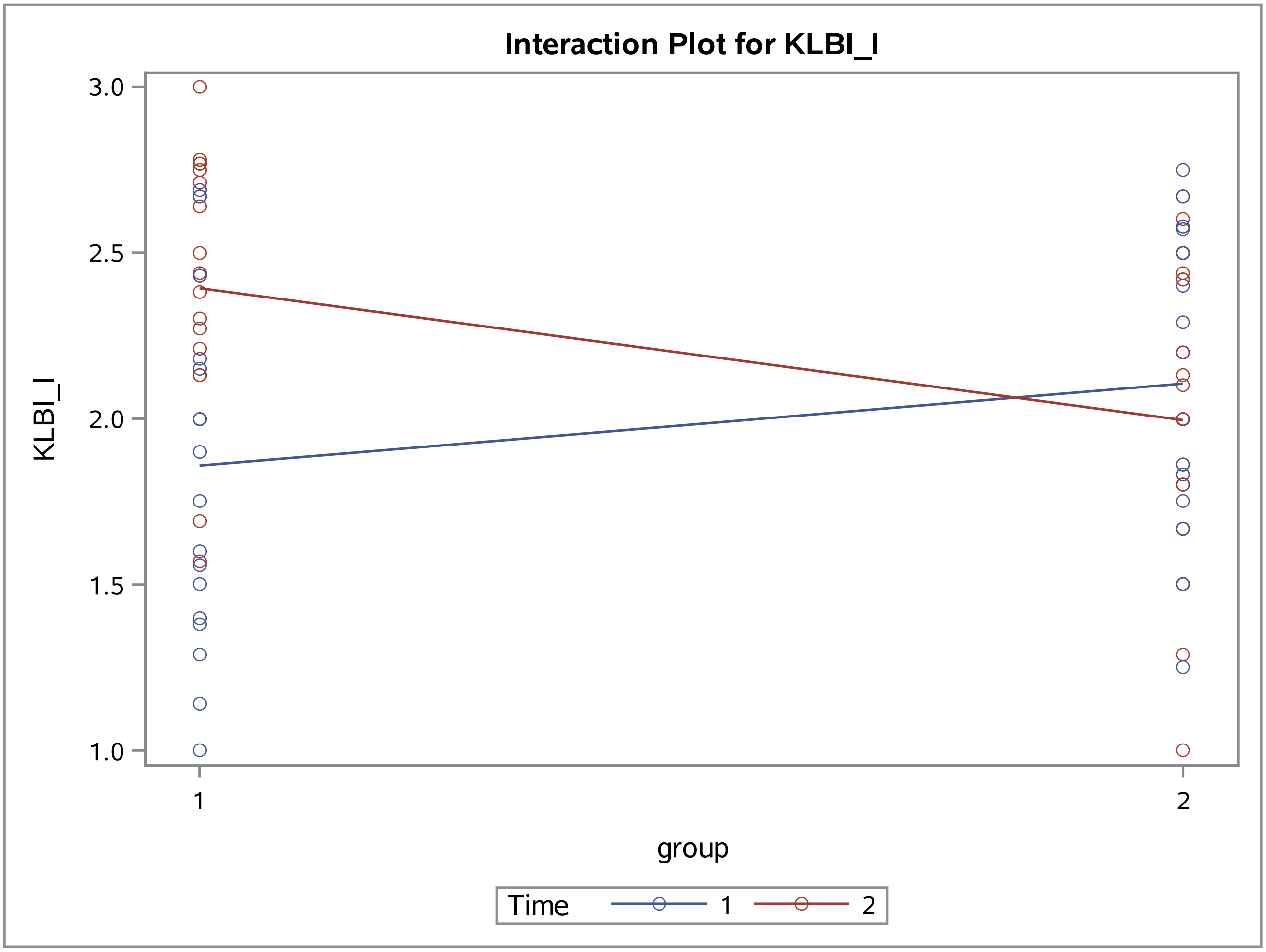

Model Information	
**Data Set**	WORK.JUNG_DATA2
**Dependent Variable**	PHQ_sum
**Covariance Structure**	Unstructured
**Subject Effect**	Subjectid
**Estimation Method**	REML
**Residual Variance Method**	None
**Fixed Effects SE Method**	Model-Based
**Degrees of Freedom Method**	Between-Within

Class Level Information

Class	Levels	Values
**Subjectid**	41	1 2 4 5 6 7 8 10 11 12 13 14 15 16 17 18 19 22 23 24 25 26 27 28 29 30 31 32 33 34 36 37 39 40 41 42 43 44 45 46 50
**group**	2	1 2
**Time**	2	1 2

Dimensions	
**Covariance Parameters**	3
**Columns in X**	9
**Columns in Z**	0
**Subjects**	41
**Max Obs per Subject**	2

Number of Observations	
**Number of Observations Read**	82
**Number of Observations Used**	82
**Number of Observations Not Used**	0

Iteration History			
**Iteration**	**Evaluations**	**-2 Res Log Like**	**Criterion**
**0**	1	485.15026194	
**1**	1	460.44725040	0.00000000

Convergence criteria met.

Covariance Parameter Estimates		
**Cov Parm**	**Subject**	**Estimate**
**UN(1,1)**	Subjectid	28.8617
**UN(2,1)**	Subjectid	16.8783
**UN(2,2)**	Subjectid	21.5583

Fit Statistics	
**-2 Res Log Likelihood**	460.4
**AIC (Smaller is Better)**	466.4
**AICC (Smaller is Better)**	466.8
**BIC (Smaller is Better)**	471.6

Null Model Likelihood Ratio Test		
**DF**	**Chi-Square**	**Pr > ChiSq**
2	24.70	<.0001

Type 3 Tests of Fixed Effects				
Effect	Num DF	Den DF	F Value	Pr > F
group	1	39	0.30	0.5881
Time	1	39	0.00	0.9466
group*Time	1	39	14.23	0.0005

Class Level Information		
Class	Levels	Values
group	2	1 2
Time	2	1 2

Number of Observations Read	82
Number of Observations Used	82

Dependent Variable: PHQ_sum

Source	DF	Sum of Squares	Mean Square	F Value	Pr > F
Model	3	132.117225	44.039075	1.75	0.1643
Error	78	1966.382775	25.210036		
Corrected Total	81	2098.500000			

R-Square	Coeff Var	Root MSE	PHQ_sum Mean
0.062958	91.29018	5.020960	5.500000

Overall Noncentrality	
Min Var Unbiased Estimate	2.1063
Low MSE Estimate	2.0509
95% Confidence Limits	(0,15.541)

Proportion of Variation Accounted for	
Eta-Square	0.06
Omega-Square	0.03
95% Confidence Limits	(0.00,0.16)

Dependent Variable: PHQ_sum

Source	DF	Type I SS	Mean Square	F Value	Pr > F	Noncentrality Parameter Min Var Unbiased Estimate	Low MSE Estimate	95% Confidence Limits	
group	1	12.5550239	12.5550239	0.50	0.4825	-0.515	-0.501	0	7.07
Time	1	0.9878049	0.9878049	0.04	0.8436	-0.962	-0.937	0	3.72
group*Time	1	118.5743961	118.5743961	4.70	0.0331	3.583	3.489	0	17.23

Source	Total Variation Accounted For: Semipartial Eta-Square	Semipartial Omega-Square	Conservative 95% Confidence Limits		Partial Variation Accounted For: Partial Eta-Square	Partial Omega-Square	95% Confidence Limits	
group	0.0060	-0.0060	0.0000	0.0787	0.0063	-0.0062	0.0000	0.0794
Time	0.0005	-0.0114	0.0000	0.0429	0.0005	-0.0119	0.0000	0.0433
group*Time	0.0565	0.0440	0.0000	0.1750	0.0569	0.0432	0.0000	0.1737

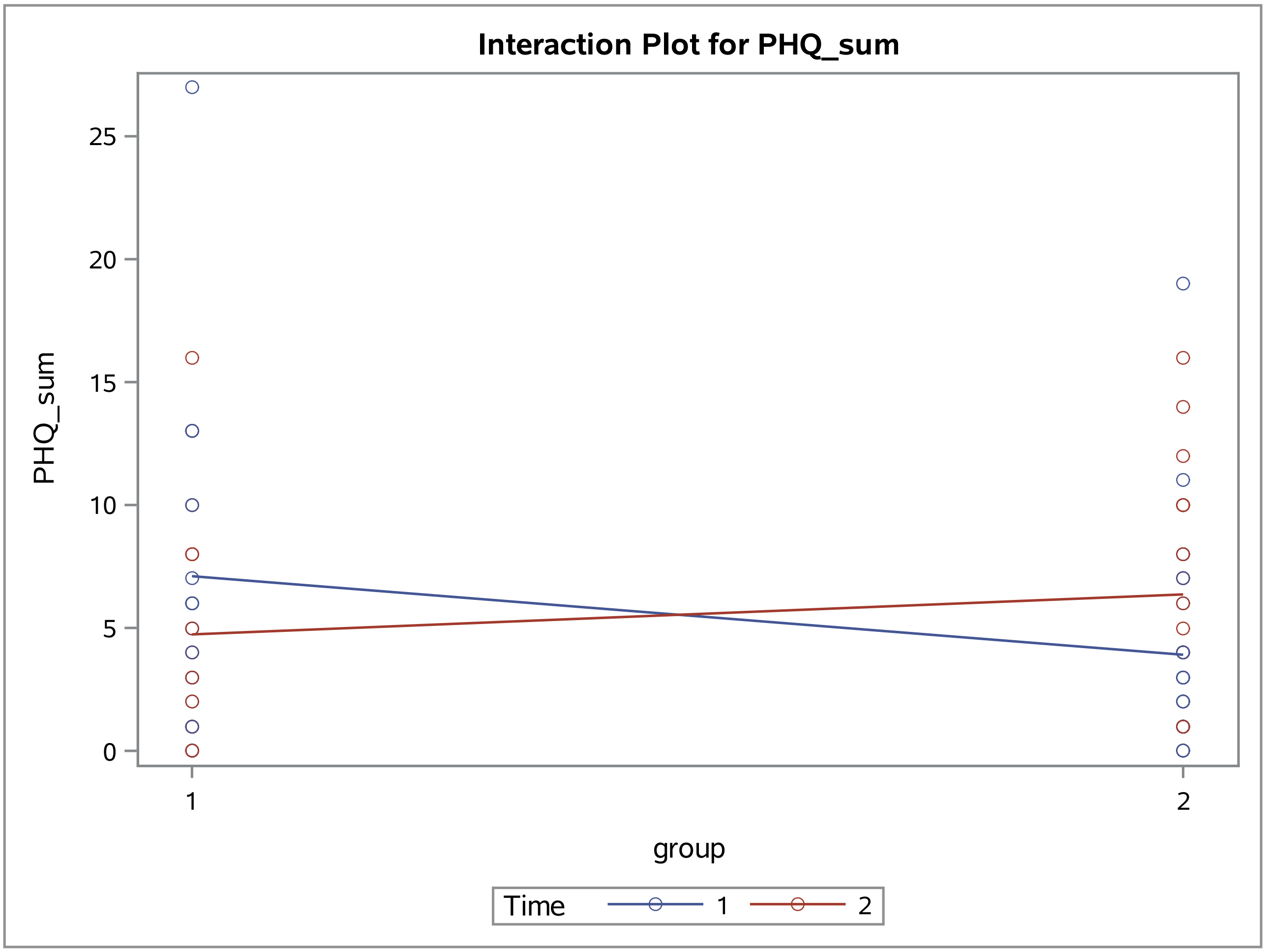

Model Information	
Data Set	WORK.JUNG_DATA2
Dependent Variable	SAS_sum
Covariance Structure	Unstructured
Subject Effect	Subjectid

Model Information	
**Estimation Method**	REML
**Residual Variance Method**	None
**Fixed Effects SE Method**	Model-Based
**Degrees of Freedom Method**	Between-Within

Class Level Information		
**Class**	**Levels**	**Values**
**Subjectid**	41	1 2 4 5 6 7 8 10 11 12 13 14 15 16 17 18 19 22 23 24 25 26 27 28 29 30 31 32 33 34 36 37 39 40 41 42 43 44 45 46 50
**group**	2	1 2
**Time**	2	1 2

Dimensions	
**Covariance Parameters**	3
**Columns in X**	9
**Columns in Z**	0
**Subjects**	41
**Max Obs per Subject**	2

Number of Observations	
**Number of Observations Read**	82
**Number of Observations Used**	82
**Number of Observations Not Used**	0

Iteration History			
**Iteration**	**Evaluations**	**-2 Res Log Like**	**Criterion**
**0**	1	535.68864501	
**1**	1	522.65481699	0.00000000

Convergence criteria met.

Covariance Parameter Estimates		
**Cov Parm**	**Subject**	**Estimate**
**UN(1,1)**	Subjectid	59.3616
**UN(2,1)**	Subjectid	23.1296
**UN(2,2)**	Subjectid	37.0202

Fit Statistics	
-2 Res Log Likelihood	522.7
AIC (Smaller is Better)	528.7
AICC (Smaller is Better)	529.0
BIC (Smaller is Better)	533.8

Null Model Likelihood Ratio Test		
DF	Chi-Square	Pr > ChiSq
2	13.03	0.0015

Type 3 Tests of Fixed Effects				
Effect	Num DF	Den DF	F Value	Pr > F
group	1	39	0.98	0.3295
Time	1	39	0.38	0.5435
group*Time	1	39	7.09	0.0112

Class Level Information		
Class	Levels	Values
group	2	1 2
Time	2	1 2

Number of Observations Read	82
Number of Observations Used	82

Dependent Variable: SAS_sum

Source	DF	Sum of Squares	Mean Square	F Value	Pr > F
Model	3	251.658828	83.886276	1.74	0.1656
Error	78	3758.889952	48.190897		
Corrected Total	81	4010.548780			

R-Square	Coeff Var	Root MSE	SAS_sum Mean
0.062749	18.90539	6.941966	36.71951

Overall Noncentrality	
Min Var Unbiased Estimate	2.0882
Low MSE Estimate	2.0333
95% Confidence Limits	(0,15.504)

Dependent Variable: SAS_sum

Proportion of Variation Accounted for	
**Eta-Square**	0.06
**Omega-Square**	0.03
**95% Confidence Limits**	(0.00,0.16)

Source	DF	Type I SS	Mean Square	F Value	Pr > F	Noncentrality Parameter: Min Var Unbiased Estimate	Noncentrality Parameter: Low MSE Estimate	Noncentrality Parameter: 95% Confidence Limits	
**group**	1	69.5511728	69.5511728	1.44	0.2333	0.406	0.396	0	10.03
**Time**	1	4.4024390	4.4024390	0.09	0.7633	-0.911	-0.887	0	4.64
**group*Time**	1	177.7052165	177.7052165	3.69	0.0585	2.593	2.525	0	15.19

Source	Total Variation Accounted For: Semipartial Eta-Square	Total Variation Accounted For: Semipartial Omega-Square	Total Variation Accounted For: Conservative 95% Confidence Limits		Partial Variation Accounted For: Partial Eta-Square	Partial Variation Accounted For: Partial Omega-Square	Partial Variation Accounted For: 95% Confidence Limits	
**group**	0.0173	0.0053	0.0000	0.1081	0.0182	0.0054	0.0000	0.1089
**Time**	0.0011	-0.0108	0.0000	0.0530	0.0012	-0.0112	0.0000	0.0535
**group*Time**	0.0443	0.0319	0.0000	0.1566	0.0451	0.0317	0.0000	0.1563

Dependent Variable: SAS_sum

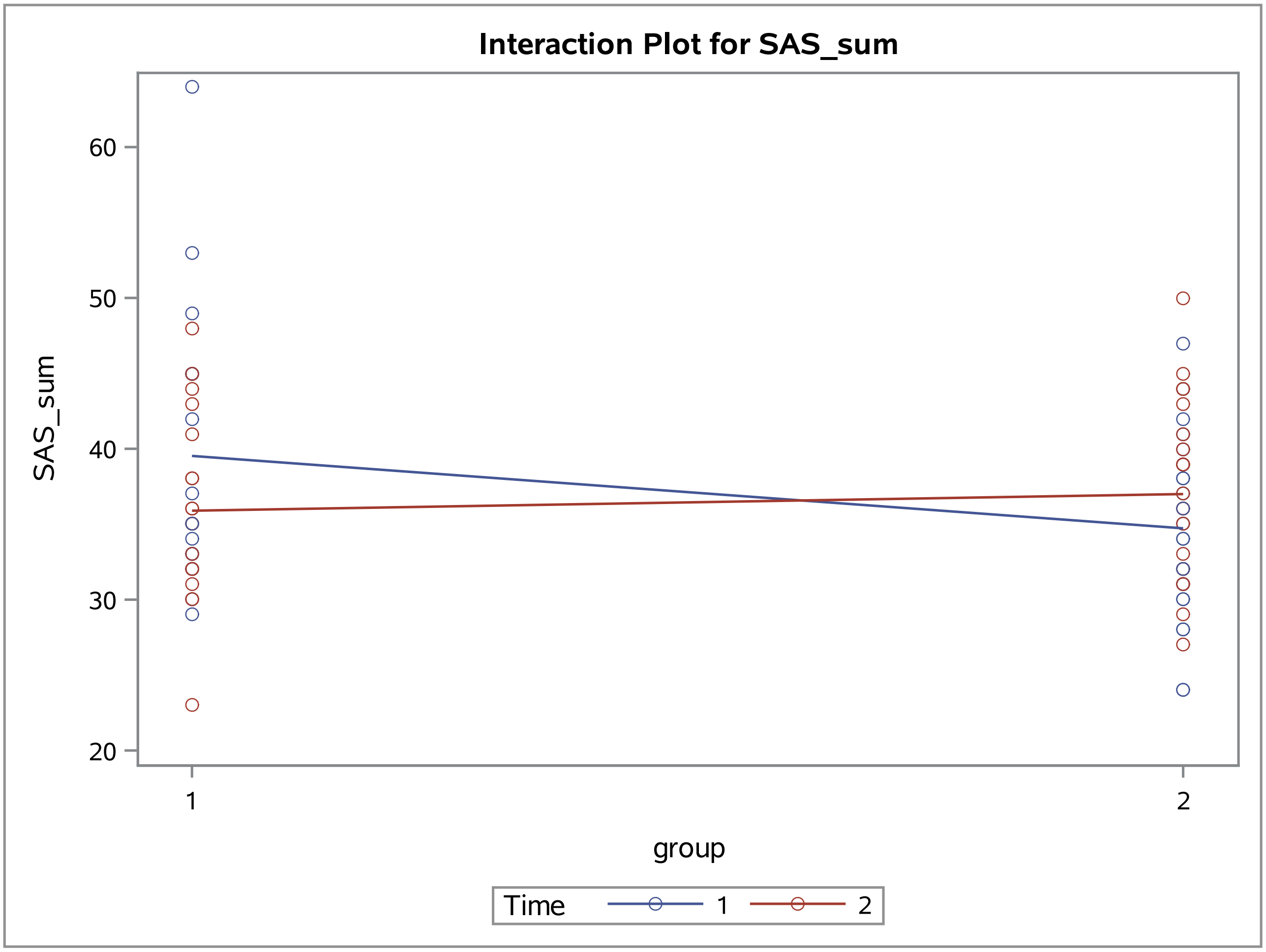

Model Information	
**Data Set**	WORK.JUNG_DATA2
**Dependent Variable**	ISI_sum
**Covariance Structure**	Unstructured
**Subject Effect**	Subjectid
**Estimation Method**	REML
**Residual Variance Method**	None
**Fixed Effects SE Method**	Model-Based
**Degrees of Freedom Method**	Between-Within

Class Level Information

Class	Levels	Values
**Subjectid**	41	1 2 4 5 6 7 8 10 11 12 13 14 15 16 17 18 19 22 23 24 25 26 27 28 29 30 31 32 33 34 36 37 39 40 41 42 43 44 45 46 50
**group**	2	1 2
**Time**	2	1 2

Dimensions	
Covariance Parameters	3
Columns in X	9
Columns in Z	0
Subjects	41
Max Obs per Subject	2

Number of Observations	
Number of Observations Read	82
Number of Observations Used	82
Number of Observations Not Used	0

Iteration History			
Iteration	Evaluations	-2 Res Log Like	Criterion
0	1	517.49945462	
1	1	492.39385682	0.00000000

Convergence criteria met.

Covariance Parameter Estimates		
Cov Parm	Subject	Estimate
UN(1,1)	Subjectid	43.5318
UN(2,1)	Subjectid	25.7428
UN(2,2)	Subjectid	32.8025

Fit Statistics	
-2 Res Log Likelihood	492.4
AIC (Smaller is Better)	498.4
AICC (Smaller is Better)	498.7
BIC (Smaller is Better)	503.5

Null Model Likelihood Ratio Test		
DF	Chi-Square	Pr > ChiSq
2	25.11	<.0001

Type 3 Tests of Fixed Effects				
Effect	Num DF	Den DF	F Value	Pr > F
group	1	39	0.66	0.4209
Time	1	39	2.08	0.1569
group*Time	1	39	31.77	<.0001

Class Level Information		
Class	Levels	Values
group	2	1 2
Time	2	1 2

Number of Observations Read	82
Number of Observations Used	82

Dependent Variable: ISI_sum

Source	DF	Sum of Squares	Mean Square	F Value	Pr > F
Model	3	450.242210	150.080737	3.93	0.0114
Error	78	2977.038278	38.167157		
Corrected Total	81	3427.280488			

R-Square	Coeff Var	Root MSE	ISI_sum Mean
0.131370	65.87679	6.177957	9.378049

Overall Noncentrality	
Min Var Unbiased Estimate	8.4941
Low MSE Estimate	8.2706
95% Confidence Limits	(0.6345,27.29)

Proportion of Variation Accounted for	
Eta-Square	0.13
Omega-Square	0.10
95% Confidence Limits	(0.01,0.25)

Dependent Variable: ISI_sum

Source	DF	Type I SS	Mean Square	F Value	Pr > F	Noncentrality Parameter: Min Var Unbiased Estimate	Noncentrality Parameter: Low MSE Estimate	Noncentrality Parameter: 95% Confidence Limits	
group	1	42.2924495	42.2924495	1.11	0.2957	0.0797	0.0776	0.0	9.09
Time	1	13.2804878	13.2804878	0.35	0.5570	-0.6610	-0.6436	0.0	6.43
group*Time	1	394.6692730	394.6692730	10.34	0.0019	9.0754	8.8366	1.4	27.35

Source	Total Variation Accounted For: Semipartial Eta-Square	Total Variation Accounted For: Semipartial Omega-Square	Total Variation Accounted For: Conservative 95% Confidence Limits		Partial Variation Accounted For: Partial Eta-Square	Partial Variation Accounted For: Partial Omega-Square	Partial Variation Accounted For: 95% Confidence Limits	
group	0.0123	0.0012	0.0000	0.0967	0.0140	0.0013	0.0000	0.0998
Time	0.0039	-0.0072	0.0000	0.0708	0.0044	-0.0080	0.0000	0.0727
group*Time	0.1152	0.1029	0.0171	0.2508	0.1171	0.1023	0.0168	0.2501

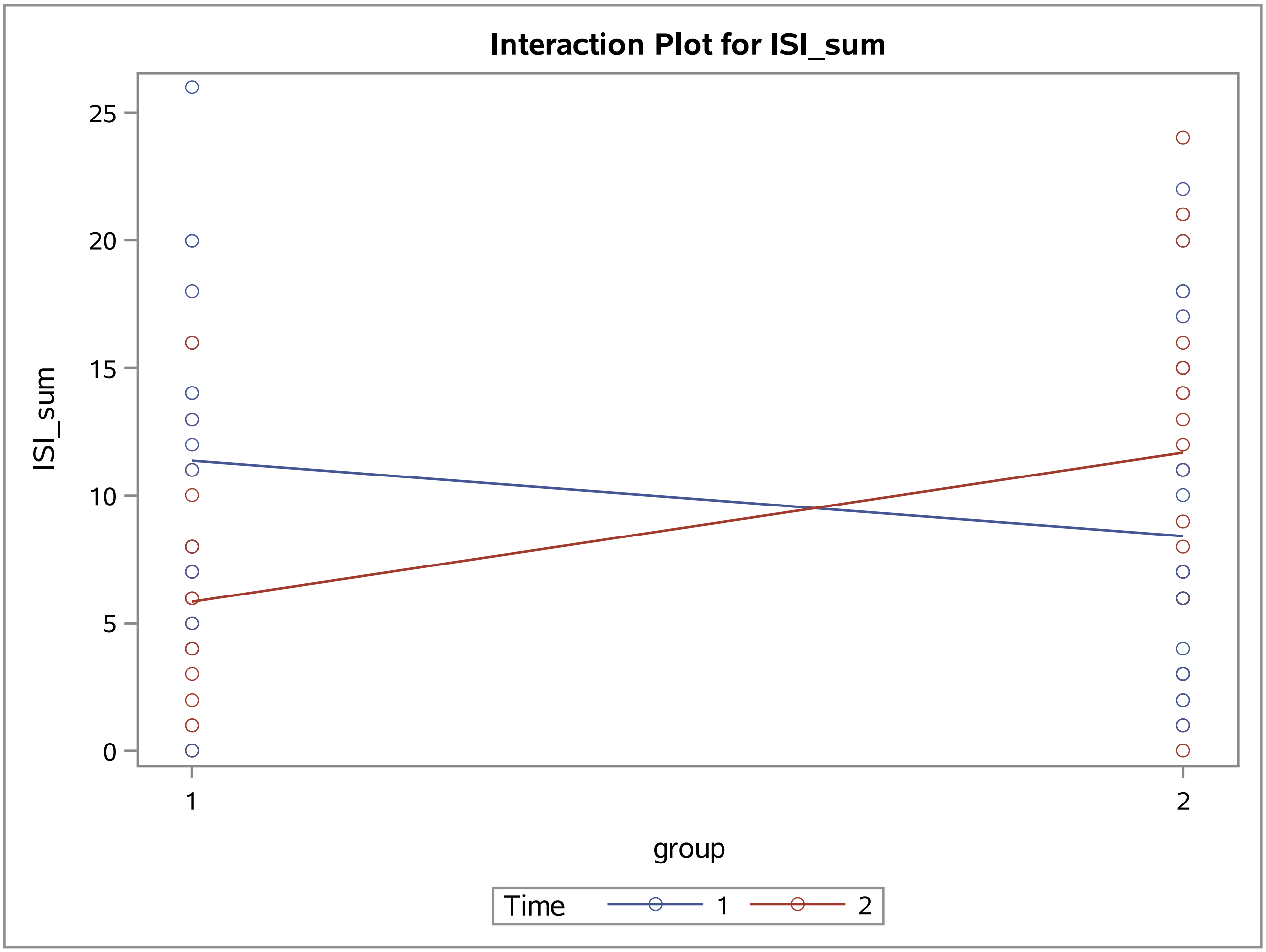

Model Information	
Data Set	WORK.JUNG_DATA2
Dependent Variable	MSBS_sum
Covariance Structure	Unstructured
Subject Effect	Subjectid

Model Information	
**Estimation Method**	REML
**Residual Variance Method**	None
**Fixed Effects SE Method**	Model-Based
**Degrees of Freedom Method**	Between-Within

Class Level Information		
**Class**	**Levels**	**Values**
**Subjectid**	41	1 2 4 5 6 7 8 10 11 12 13 14 15 16 17 18 19 22 23 24 25 26 27 28 29 30 31 32 33 34 36 37 39 40 41 42 43 44 45 46 50
**group**	2	1 2
**Time**	2	1 2

Dimensions	
**Covariance Parameters**	3
**Columns in X**	9
**Columns in Z**	0
**Subjects**	41
**Max Obs per Subject**	2

Number of Observations	
**Number of Observations Read**	82
**Number of Observations Used**	82
**Number of Observations Not Used**	0

Iteration History			
**Iteration**	**Evaluations**	**-2 Res Log Like**	**Criterion**
**0**	1	577.20354691	
**1**	1	558.10624595	0.00000000

Convergence criteria met.

Covariance Parameter Estimates		
**Cov Parm**	**Subject**	**Estimate**
**UN(1,1)**	Subjectid	96.0284
**UN(2,1)**	Subjectid	49.1097
**UN(2,2)**	Subjectid	68.0853

Fit Statistics	
-2 Res Log Likelihood	558.1
AIC (Smaller is Better)	564.1
AICC (Smaller is Better)	564.4
BIC (Smaller is Better)	569.2

Null Model Likelihood Ratio Test		
DF	Chi-Square	Pr > ChiSq
2	19.10	<.0001

Type 3 Tests of Fixed Effects				
Effect	Num DF	Den DF	F Value	Pr > F
group	1	39	0.91	0.3447
Time	1	39	0.18	0.6716
group*Time	1	39	21.63	<.0001

Class Level Information		
Class	Levels	Values
group	2	1 2
Time	2	1 2

Number of Observations Read	82
Number of Observations Used	82

Dependent Variable: MSBS_sum

Source	DF	Sum of Squares	Mean Square	F Value	Pr > F
Model	3	852.052398	284.017466	3.46	0.0203
Error	78	6400.435407	82.056864		
Corrected Total	81	7252.487805			

R-Square	Coeff Var	Root MSE	MSBS_sum Mean
0.117484	34.16739	9.058524	26.51220

Overall Noncentrality	
Min Var Unbiased Estimate	7.1174
Low MSE Estimate	6.9301
95% Confidence Limits	(0.161,24.902)

Dependent Variable: MSBS_sum

Proportion of Variation Accounted for	
Eta-Square	0.12
Omega-Square	0.08
95% Confidence Limits	(0.00,0.23)

						Noncentrality Parameter			
Source	DF	Type I SS	Mean Square	F Value	Pr > F	Min Var Unbiased Estimate	Low MSE Estimate	95% Confidence Limits	
group	1	119.9901972	119.9901972	1.46	0.2302	0.425	0.414	0.000	10.08
Time	1	19.5121951	19.5121951	0.24	0.6272	-0.768	-0.748	0.000	5.84
group*Time	1	712.5500058	712.5500058	8.68	0.0042	7.461	7.265	0.851	24.52

	Total Variation Accounted For				Partial Variation Accounted For			
Source	Semipartial Eta-Square	Semipartial Omega-Square	Conservative 95% Confidence Limits		Partial Eta-Square	Partial Omega-Square	95% Confidence Limits	
group	0.0165	0.0052	0.0000	0.1064	0.0184	0.0056	0.0000	0.1094
Time	0.0027	-0.0085	0.0000	0.0650	0.0030	-0.0094	0.0000	0.0665
group*Time	0.0982	0.0860	0.0104	0.2305	0.1002	0.0857	0.0103	0.2302

Dependent Variable: MSBS_sum

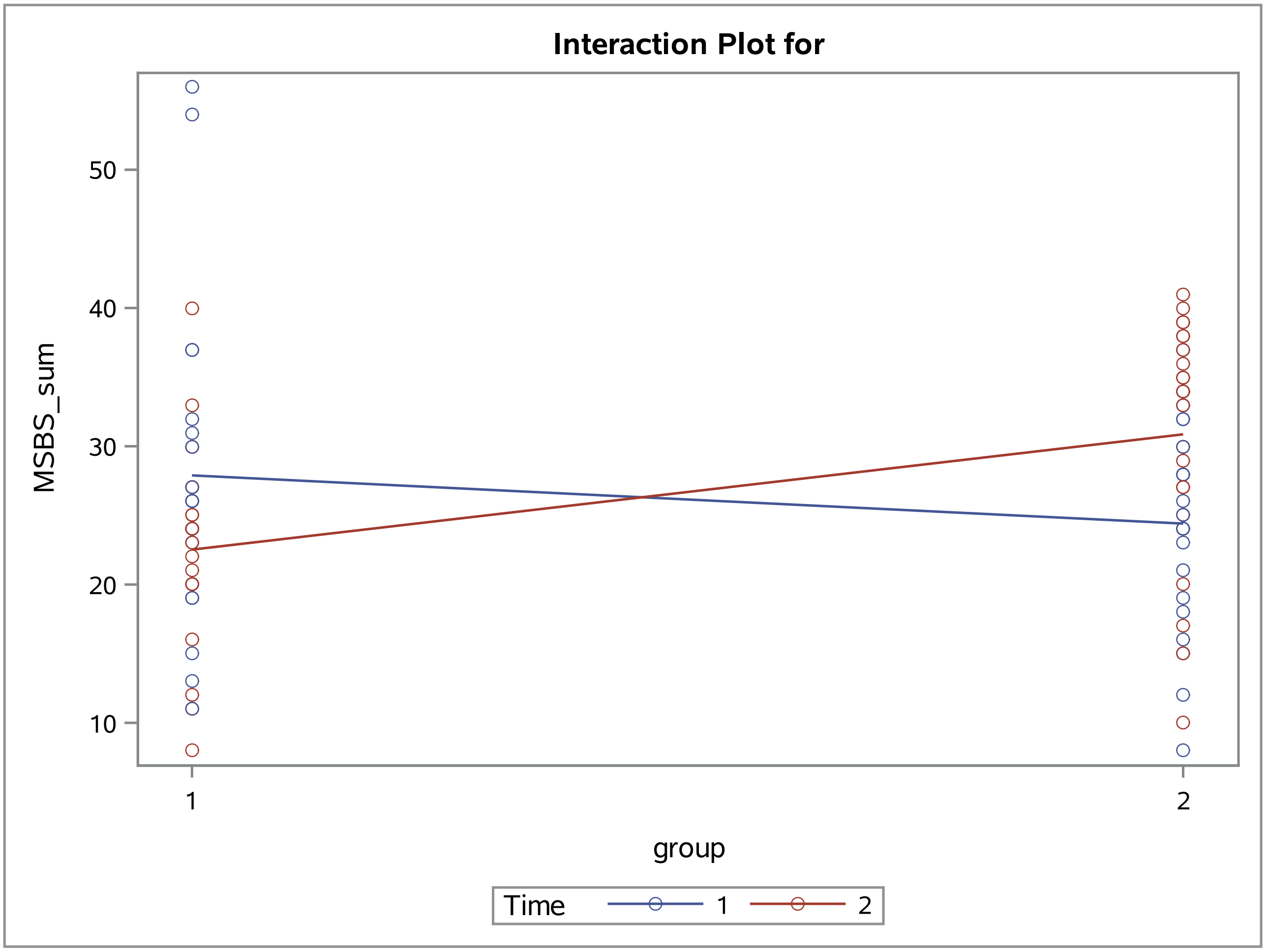

Model Information	
**Data Set**	WORK.JUNG_DATA2
**Dependent Variable**	Fear_sum
**Covariance Structure**	Unstructured
**Subject Effect**	Subjectid
**Estimation Method**	REML
**Residual Variance Method**	None
**Fixed Effects SE Method**	Model-Based
**Degrees of Freedom Method**	Between-Within

Class Level Information

Class	Levels	Values
**Subjectid**	41	1 2 4 5 6 7 8 10 11 12 13 14 15 16 17 18 19 22 23 24 25 26 27 28 29 30 31 32 33 34 36 37 39 40 41 42 43 44 45 46 50
**group**	2	1 2
**Time**	2	1 2

Dimensions	
Covariance Parameters	3
Columns in X	9
Columns in Z	0
Subjects	41
Max Obs per Subject	2

Number of Observations	
Number of Observations Read	82
Number of Observations Used	82
Number of Observations Not Used	0

Iteration History

Iteration	Evaluations	-2 Res Log Like	Criterion
0	1	514.65503808	
1	1	502.76060234	0.00000000

Convergence criteria met.

Covariance Parameter Estimates

Cov Parm	Subject	Estimate
UN(1,1)	Subjectid	40.2159
UN(2,1)	Subjectid	18.5560
UN(2,2)	Subjectid	33.3849

Fit Statistics	
-2 Res Log Likelihood	502.8
AIC (Smaller is Better)	508.8
AICC (Smaller is Better)	509.1
BIC (Smaller is Better)	513.9

Null Model Likelihood Ratio Test

DF	Chi-Square	Pr > ChiSq
2	11.89	0.0026

Type 3 Tests of Fixed Effects				
Effect	Num DF	Den DF	F Value	Pr > F
group	1	39	0.00	0.9942
Time	1	39	0.32	0.5726
group*Time	1	39	12.59	0.0010

Class Level Information		
Class	Levels	Values
group	2	1 2
Time	2	1 2

Number of Observations Read	82
Number of Observations Used	82

Dependent Variable: Fear_sum

Source	DF	Sum of Squares	Mean Square	F Value	Pr > F
Model	3	231.471817	77.157272	2.10	0.1074
Error	78	2870.430622	36.800393		
Corrected Total	81	3101.902439			

R-Square	Coeff Var	Root MSE	Fear_sum Mean
0.074623	31.24619	6.066333	19.41463

Overall Noncentrality	
Min Var Unbiased Estimate	3.1286
Low MSE Estimate	3.0463
95% Confidence Limits	(0,17.574)

Proportion of Variation Accounted for	
Eta-Square	0.07
Omega-Square	0.04
95% Confidence Limits	(0.00,0.18)

Dependent Variable: Fear_sum

Source	DF	Type I SS	Mean Square	F Value	Pr > F	Noncentrality Parameter			
						Min Var Unbiased Estimate	Low MSE Estimate	95% Confidence Limits	
group	1	0.0029175	0.0029175	0.00	0.9929	-1.000	-0.974	0.000	0.00
Time	1	1.7560976	1.7560976	0.05	0.8277	-0.954	-0.928	0.000	3.92
group*Time	1	229.7128020	229.7128020	6.24	0.0146	5.082	4.948	0.209	20.15

Source	Total Variation Accounted For				Partial Variation Accounted For			
	Semipartial Eta-Square	Semipartial Omega-Square	Conservative 95% Confidence Limits		Partial Eta-Square	Partial Omega-Square	95% Confidence Limits	
group	0.0000	-0.0117	0.0000	0.0000	0.0000	-0.0123	0.0000	0.0000
Time	0.0006	-0.0112	0.0000	0.0451	0.0006	-0.0117	0.0000	0.0457
group*Time	0.0741	0.0615	0.0030	0.1995	0.0741	0.0601	0.0025	0.1973

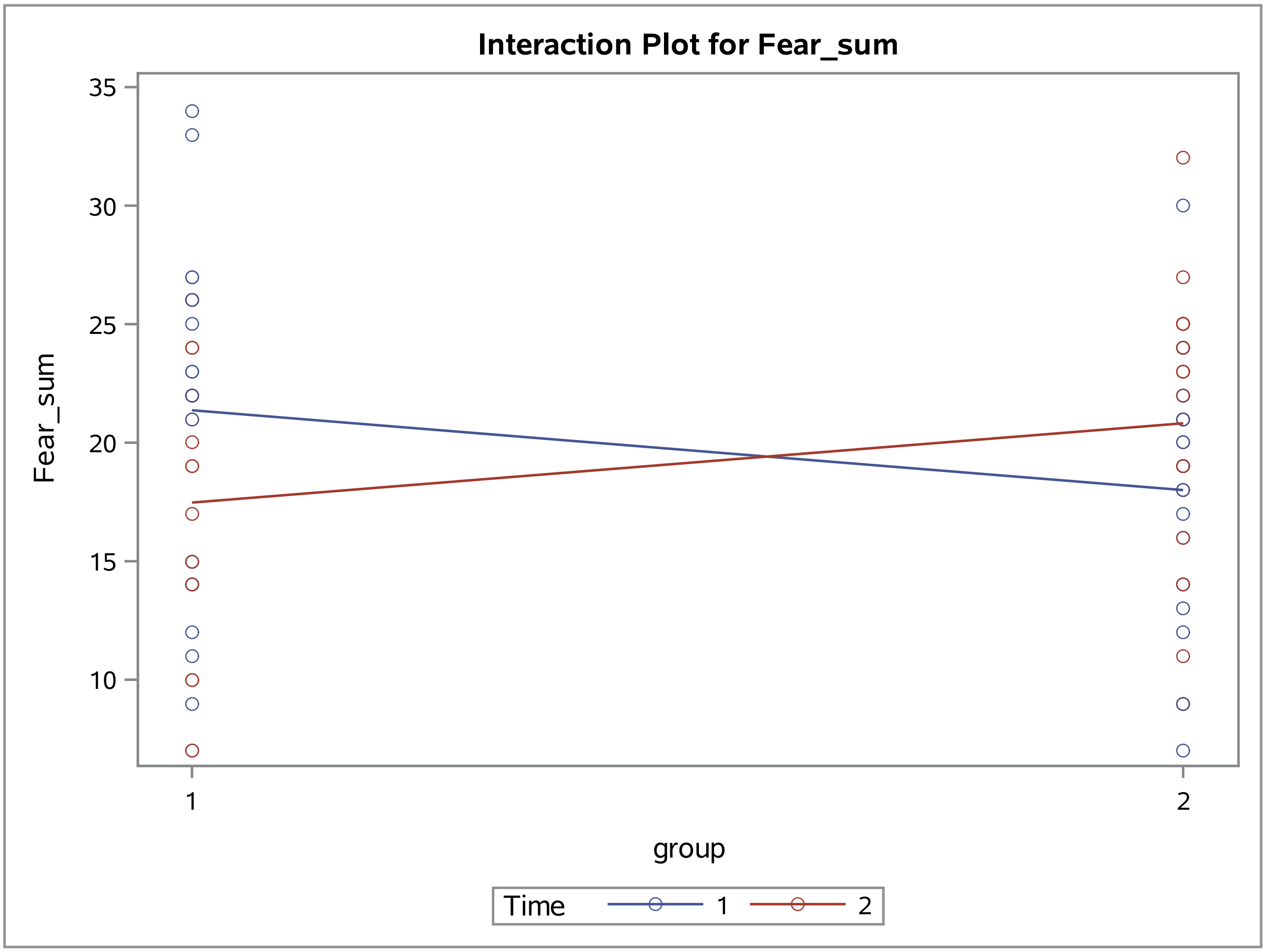

Model Information	
Data Set	WORK.JUNG_DATA2
Dependent Variable	WHOQOL_sum
Covariance Structure	Unstructured
Subject Effect	Subjectid

Model Information	
Estimation Method	REML
Residual Variance Method	None
Fixed Effects SE Method	Model-Based
Degrees of Freedom Method	Between-Within

Class Level Information		
Class	Levels	Values
Subjectid	41	1 2 4 5 6 7 8 10 11 12 13 14 15 16 17 18 19 22 23 24 25 26 27 28 29 30 31 32 33 34 36 37 39 40 41 42 43 44 45 46 50
group	2	1 2
Time	2	1 2

Dimensions	
Covariance Parameters	3
Columns in X	9
Columns in Z	0
Subjects	41
Max Obs per Subject	2

Number of Observations	
Number of Observations Read	82
Number of Observations Used	82
Number of Observations Not Used	0

Iteration History			
Iteration	Evaluations	-2 Res Log Like	Criterion
0	1	584.09986263	
1	1	571.98980683	0.00000000

Convergence criteria met.

Covariance Parameter Estimates		
Cov Parm	Subject	Estimate
UN(1,1)	Subjectid	76.4664
UN(2,1)	Subjectid	44.4002
UN(2,2)	Subjectid	102.82

Fit Statistics	
-2 Res Log Likelihood	572.0
AIC (Smaller is Better)	578.0
AICC (Smaller is Better)	578.3
BIC (Smaller is Better)	583.1

Null Model Likelihood Ratio Test		
DF	Chi-Square	Pr > ChiSq
2	12.11	0.0023

Type 3 Tests of Fixed Effects				
Effect	Num DF	Den DF	F Value	Pr > F
group	1	39	0.54	0.4655
Time	1	39	8.08	0.0071
group*Time	1	39	21.28	<.0001

Class Level Information		
Class	Levels	Values
group	2	1 2
Time	2	1 2

Number of Observations Read	82
Number of Observations Used	82

Dependent Variable: WHOQOL_sum

Source	DF	Sum of Squares	Mean Square	F Value	Pr > F
Model	3	1320.892111	440.297370	4.91	0.0035
Error	78	6992.095694	89.642252		
Corrected Total	81	8312.987805			

R-Square	Coeff Var	Root MSE	WHOQOL_sum Mean
0.158895	11.00769	9.467959	86.01220

Overall Noncentrality	
Min Var Unbiased Estimate	11.357
Low MSE Estimate	11.058
95% Confidence Limits	(1.7659,32.111)

Dependent Variable: WHOQOL_sum

Proportion of Variation Accounted for	
Eta-Square	0.16
Omega-Square	0.13
95% Confidence Limits	(0.02,0.28)

						Noncentrality Parameter			
Source	DF	Type I SS	Mean Square	F Value	Pr > F	Min Var Unbiased Estimate	Low MSE Estimate	95% Confidence Limits	
group	1	72.8323025	72.8323025	0.81	0.3702	-0.208	-0.203	0.00	8.19
Time	1	285.4756098	285.4756098	3.18	0.0782	2.103	2.048	0.00	14.13
group*Time	1	962.5841989	962.5841989	10.74	0.0016	9.463	9.214	1.54	28.02

	Total Variation Accounted For				Partial Variation Accounted For			
Source	Semipartial Eta-Square	Semipartial Omega-Square	Conservative 95% Confidence Limits		Partial Eta-Square	Partial Omega-Square	95% Confidence Limits	
group	0.0088	-0.0020	0.0000	0.0873	0.0103	-0.0023	0.0000	0.0908
Time	0.0343	0.0233	0.0000	0.1403	0.0392	0.0260	0.0000	0.1470
group*Time	0.1158	0.1039	0.0174	0.2516	0.1210	0.1062	0.0184	0.2547

Dependent Variable: WHOQOL_sum

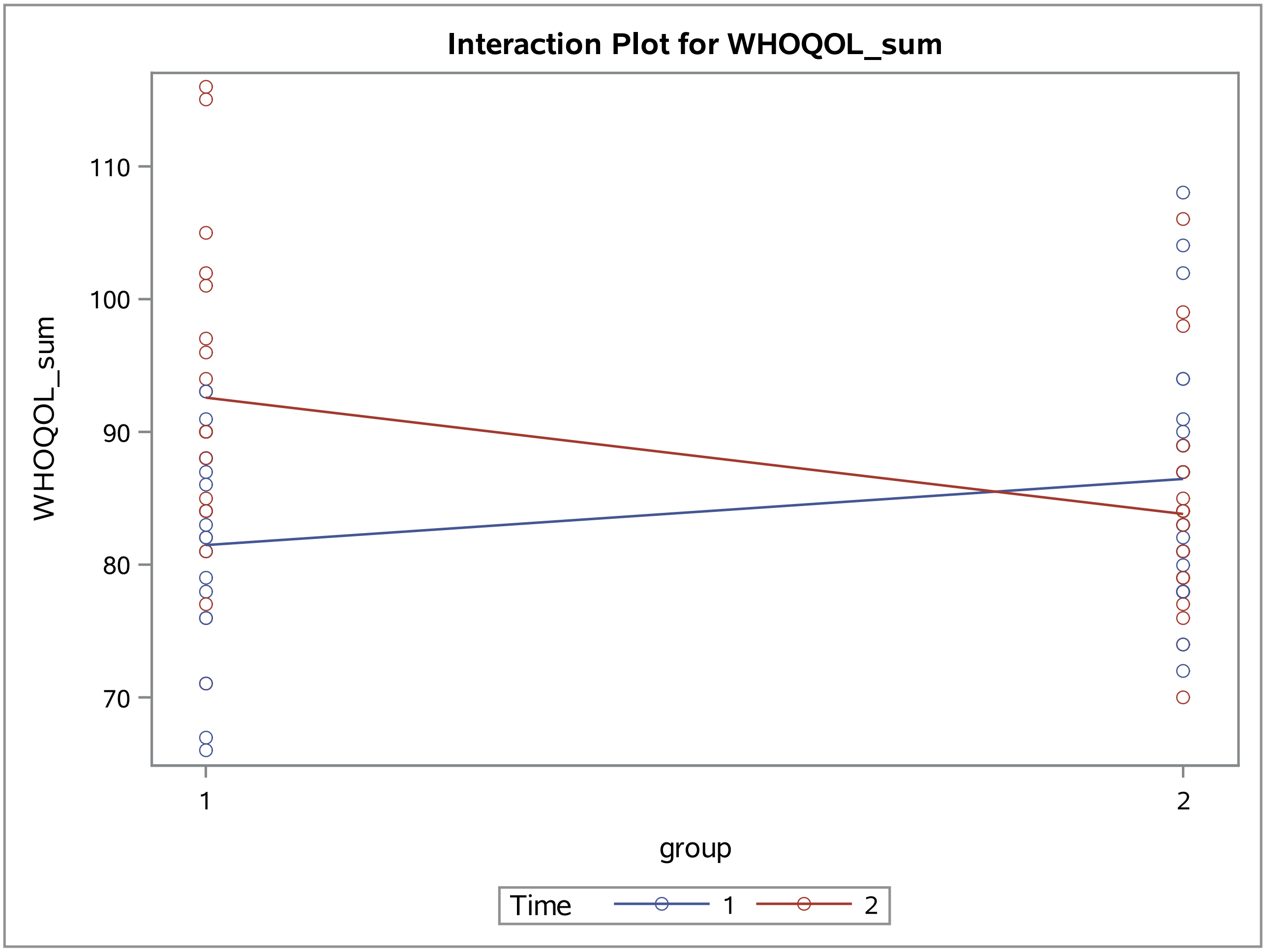

Supplement: S1 File — (PDF) [file pone.0287118.s002.pdf]
