# Supplementary material for: Effects of a time-use intervention in isolated patients with coronavirus disease 2019: A randomized controlled study
Source: PLoS One. 2023 Jun 23;18(6):e0287118. doi: 10.1371/journal.pone.0287118 (PMC10289446; doi:10.1371/journal.pone.0287118)
Supplement: S2 File — (PDF) [file pone.0287118.s003.pdf]

Tile: Effects of Time-use Intervention on Occupational Balance, Mental Health, and Quality of Life in Isolated Patients with Coronavirus Disease 2019

1. Background

COVID 19 started in Wuhan, China in December 2019 and spread worldwide. WHO has declared COVID-19 a public health emergency of international concern (PHEIC). In Korea, strict measures are being taken to isolate patients diagnosed or suspected of having COVID-19 due to a prolonged national emergency with a rapid increase in confirmed cases (WHO, 2020; Central Disaster and Safety Countermeasure Headquarters, 2020). Among COVID-19 patients, 19% of patients show more than severe physical symptoms such as respiratory failure and septic shock, and 81% of patients show mild symptoms, which is more than severe patients (Wu Z et al., 2020). COVID-19 patients complain of anxiety and depression along with stressors such as long isolation period, fear of infection, frustration, boredom, inappropriate information, financial loss, and stigma, as well as physical health problems (Fan et al., 2020; Jiménez -Pavón, D et al., 2020). Post-traumatic stress disorder, depression, anxiety, insomnia, and decreased quality of life were consistently observed in survivors of severe acute respiratory syndrome (SARS) and Middle East respiratory syndrome (MERS) 1 year after discharge. From this, it can be assumed that these symptoms will continue in COVID-19 patients (Lei et al., 2008; Stephen et al., 2020).

Currently, COVID-19 positive diagnoses in Korea are isolated in living centers and dedicated hospitals. Isolation greatly aids in shrinking the virus and is the best option to prevent infection, but there are several downsides to isolation (Jiménez et al., 2020). Isolation causes changes in lifestyle, a complex measure of health that includes many factors such as personal values, lifestyle, and culture (Mandel, 1999; Kim, 2016), and also destroys the balance of meaningful occupation (Kamalakannan & Chakraborty, 2020). It can be a risk

factor for the development of physical health and psychological diseases by experiencing stress such as boredom, fear, anxiety, loneliness, excessive worry, and depression (Abad et al., 2010; Jiménez et al., 2020; Soheili, et al., 2020).

For disaster victims, participation in meaningful work can play an important role in reducing stress, alleviating negative reactions, and promoting physical and mental recovery, leading to a worthwhile life and return to original roles (Yamkovenko, 2008; Smith & Scaffa, 2013; Kamalakannan & Chakraborty., 2020). In addition, the National Trauma Center recommends activities (exercise, activity, regular meals, positive attitude advice) during the day for COVID-19 patients (National Trauma Center, 2020). Therefore, it will be important for health to maintain a occupational balance by maintaining an active lifestyle without lifestyle changes or disruptions (Amin, K. P et al., 2020; Jiménez-Pavón, D et al., 2020).

Occupational balance, defined as the organization of daily activities, enables health and well-being by allowing various participations such as work, household management, childcare, and leisure and rest activities (Wilcock et al., 1997). Time use is closely related to occupational balance as it is fundamental to how people organize and structure their daily lives (Edgelow & Krupa, 2011), and understanding how people spend their time is a particularly useful approach to occupational balance and engagement studies (Pentland & McColl, 1999).

Time use intervention is an intervention to properly allocate time within the occupation area to maintain occupational balance and is based on the Model of Human Occupation (MOHO) (Jeon, 2011). It showed positive effects on their depression reduction, quality of life improvement, self-esteem, and life satisfaction for Spinal cord injury patients, stroke patients, psychiatric patients, elderly people, women, etc. (Kim Young-geun, 2010; Kim Ji-hoon, 2014). Youngju Park et al., 2015; Seonghyun Ryu et al., 2020 Edgelow & Krupa., 2011; Gutman et al., 2020).

In the case of COVID-19 patients, changes in lifestyle, occupation imbalance, and mental health problems have been reported due to environmental and psychological factors, but studies applying interventions to these are lacking. Therefore, occupational time use interventions were conducted for isolated patients with COVID-19 to analyze their effects on occupational balance, mental health, and quality of life.

2. Purpose of study

In this study, we aimed to investigate the effects of time use interventions on occupational balance, mental health, and quality of life of patients who were diagnosed for COVID-19. A time use intervention is scheduling a routine to promote time engaged in meaningful activities. An assessment tool to measure occupational balance, mental health, and quality of life before and after the intervention is conducted using a Google online survey form. The results of this study will be the basis for interventions that help occupational balance, mental health, and quality of life for COVID-19 patients, and will be used as clinical evidence for patients' coping with isolation experiences.

3. Research organization: Gyeonggi Provincial Medical Center

4. Research support organization: None

5. Research period: 8 months from approval date

6. Research participants

1) Inclusion criteria

- Persons who isolated in hospital due to diagnosed with COVID-19

- Persons classified as mild by medical staff

- Those without underlying respiratory diseases

- Oxygen saturation measured over 95% and hemodynamically stable

- Those who do not have prominent pneumonia findings on chest radiography

- Adults over 18 years of age

- Persons who understands the purpose of the study and voluntarily agrees to participate in the study

2) Exclusion criteria

- Those who are not medically stable

- Those who have difficulty using Korean or have communication problems

7. Expected number of participants

A total of 50 subjects were calculated through the analysis of previous studies.

- A study on the effects of relaxation activities on anxiety and sleep quality in patients with COVID-19

Liu, K et al., 2020 – 51 COVID patients

- A study on occupational therapy (interview, activity-based intervention) for patients with psychiatric symptoms

Hoshii et al., 2013 - 59 patients with psychiatric symptoms

Shimada et al., 2016 – 51 psychiatric patients

8. Recruitment of participants

1) Patients admitted to the hospital were provided with sufficient information about the study over the phone, and consent was obtained to participate in the study.

2) Protection of personal privacy and confidentiality

- Personal information collected from subjects participating in this research will be used for up to one year after the research ends, and the collected information will be properly managed in accordance with the Personal Information Protection Act.
- The personal information is not provided to anyone other than the researcher. After the research is completed, personal information will be kept for 3 years after the research is completed and will be discarded thereafter.
- The subject's personal information is managed so that it is kept confidential, records that can be identified are assigned a management number to anonymize, and access to research-related data is prohibited except for those related to the research and will be stored in a locked place.
- In addition, even if the research results are published, the identity of the subjects will be kept confidential.

9. Consent of participants

- The researcher explained the purpose and background of the study to the subject, showed the consent form, and obtained consent from the research participant.
- Participants can withdraw consent at any time during participation, and there is no disadvantage.

10. Research method

1) Randomization of participants

- 50 subjects were randomly assigned to 25 experimental groups and 25 control groups
- In order not to cause selection bias, random assignment is used for block size (Kang Hyun., 2017). 50 subjects were randomly assigned to two groups, A and B, in blocks with an

allocation ratio of 1:1. In this study, a sample size of 50 people and block sizes of 6, 8, 10, 12, and 14 were used.

2) Intervention

* All interventions applied to study participants were performed in their own hospital room

(1) Time use intervention (scheduling)

- Conducted in the experimental group

- A total of 10 sessions for 2 weeks, 5 times a week (every day on weekdays)

- Steps 1-3 on the first day (takes about 40 minutes)

- Perform Step 4 from the second day to the last day (takes about 15 minutes)

- Procedure

Step	Program	Contents	Evidence
1	Time-use analysis	- Confirmation of occupational imbalance through K-LBI results<br>- Time usage analysis through Occupational Questionnaire	Smith et al.(1986)<br>김영근(2010)<br>김지훈(2014)
2	Occupation selection	- Think fixed time activity (measure vital signs, eating), essential activities (sleep and personal hygiene, etc.), individuals' activities (remote classes, praying, etc.)<br>- Provide a list of possible activities in the hospital room and select additional activities through an interview with the therapist.<br>- Occupation: personal hygiene, eating, physical activities, stretching and strengthening exercise, handicraft activities (punching embroidery, scratch art, overcoat painting, coloring book, knitting), other leisure activities(puzzles, reading, and etc.; calling with family, cleaning, and etc.), and etc.	Reconstruction based on 홍승표 등 (2008), Law et al (1998), and 김지훈(2014)
3	Activity assignment	- Based on Steps 1 and 2, the selected meaningful occupation is placed in meaningless time zones.	김영근(2010)<br>김지훈(2014)
4	Practice and check up	- Executed as planned, interviewed about the previous days' time use, and corrected and supplemented activities	김영근(2010)<br>박수진(2015)

(2) Provide educational materials for self-education and exercise

- Conducted on control group

- Once hospitalized, provided for 30 minutes

- Educational materials are provided along with documents required for hospitalization, and training is conducted over the phone.

: Educational materials are produced for COVID-19 patients and about stress management and stabilization techniques.

(3) Medical treatment and conservative treatment

: Isolation and conservative treatment according to COVID-19 response guidelines

: Performed identically to the experimental group and the control group by the medical staff.

3) Study procedure

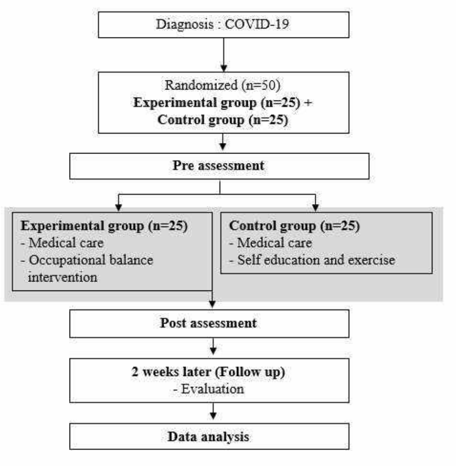

11. Outcome measurements

1) General information of research participants

- Collect information such as gender, age, and onset period through medical record information

- Evaluate occupational balance, mental health, and quality of life using the following self-report assessment tool using the form of a Google questionnaire that can be sent via text

- Evaluations are conducted when before intervention (on the first day), after intervention (on the 10th day when the intervention is completed), and after discharge (2 weeks after discharge).

2) Occupational Balance: Korean Version of Life Balance Inventory(K-LBI)

As an assessment tool for measuring Occupational balance, Matuska (2012) developed an assessment tool based on the life balance model, which Sangmi Park & Jihyeok Park (2019) translated into Korean and standardized it. It consists of 53 activities (ADL, IADL, work, play, rest and sleep, leisure, education, social participation) and 4 sub-items (health, identity, relationship, challenge). Participants response on a 5-point scale ranging from 1 (always less or more than I want) to 3 (usually as much as I want). The closer the score is to 3, the better the balance between tasks is interpreted. Cronbach's α was 0.88 for the elderly and 0.83 for adults (Park Sang-mi & Park Ji-hyeok, 2019).

3) Mental health

(1) Depression: Patient Health Questionnaire-9(PHQ-9)

As a self-report tool used to diagnose, screen, monitor, and measure the severity of depression, the assessment tool developed by Spitzer et al. (1999) was adapted and standardized by Choi Hong-seok et al. (2007). It consists of a total of 9 items and is evaluated as 'none', '2~3 days', 'more than 7 days', and 'almost every day' using a 4-point scale (Choi Hong-seok et al., 2007). Cronbach α was 0.92 (Park Seung-jin et al., 2010).

(2) Anxiety: Self-rating anxiety scale (SAS)

An assessment tool developed by William W. K. Zung in 1971 to measure the level of anxiety in patients with anxiety-related symptoms. It consists of 15 anxiety level increasing

questions and 5 anxiety reducing questions out of 20 questions, using a 4-point scale ranging from ‘almost never’ to ‘almost every day’.

(3) Sleep quality: Korean version of Insomnia severity index (ISI-K)

The ISI-K is a tool designed by Charles to assess the severity of the nighttime and daytime components of insomnia and was adapted by Yongwon Cho in 2014. It consists of a 7 items and uses a 5-point Likert scale. A score of 7 or less is interpreted as normal, 8–14 as subthreshold insomnia, 15–21 as moderate insomnia, and 22–28 as severe insomnia. Cronbach α was found to be 0.92 (Cho et al., 2014).

(4) Boredom: Multidimensional State Boredom Scale-8 (MSBS-8)

The MSBS-8 is a tool to assess participants' boredom. It was developed by abbreviating the MSBS of 29 items devised by Fahlman in 2011 into 8 items. A 7-point Likert scale is used, with higher scores reflecting greater boredom (Hunter et al., 2015).

(5) Fear of COVID-19: Fear of COVID-19 scale (FCV-19S)

The FCV-19S is a self-report assessment tool that measures fear of COVID-19. It consists of 7 items corresponding to the areas of emotional response and symptomatic expression of fear. A 5-point scale is used, with higher scores indicating greater fear of COVID-19. Cronbach α was found to be 0.82 (Ahorsu et al., 2020).

4) Quality of life: World Health Organization Quality of Life Assessment Instrument-BRIEF (Korean version of WHOQOL-BREF)

As a tool to evaluate health-related quality of life, the WHOQOL-BREF developed by the WHOQOL Group in 1998 was adapted into a Korean version in 2000. It consists of a total of 26 questions, and each question uses 5-point Likert scale. Along with overall health-related quality of life, it consists of four sub-domains: physical health domain, psychological health domain, social relationship domain, and environment domain (Whoqol Group, 1998).

Cronbach's α was 0.898, and the test-retest reliability ranged from 0.436 to 0.731 (Sung-Gil Min, Chang-Il Lee, Gwang-Il Kim, Shin-Young Seo, & Dong-Ki Kim, 2000).

12. Effect evaluation criteria and methods

When statistically analyzing changes in occupational balance, mental health, and quality of life pre, post, and after discharge, a p value of 0.05 or less is used as the criterion for effect.

13. Safety evaluation criteria and evaluation method

- This study is an intervention with sufficient evidence, and safe management is possible because the evaluation and intervention are conducted 1:1 with the therapist.
- Apart from this study, the vital signs of patients with COVID-19 are periodically measured by nurse and the patient's condition is continuously monitored through CCTV, so if the patient complains of fatigue or discomfort during or after evaluation or intervention, immediate medical treatment is possible.

14. Data analysis and statistical methods

- Descriptive analysis: General characteristics of subjects
- Analysis of pre-post evaluation and post-follow-up evaluation scores of each evaluation

: Analyze pre-post differences in the same sample using paired t-test or Wilcoxon signed-rank test.

: Analyze the difference between the experimental group and the control group using independent t-test or Mann-Whitney u test.

- Analyze the average score of pre-post-follow-up evaluation of each evaluation

: using ANOVA or Kruskal-Willi's test.

15. Research Schedule

* After the date of IRB approval ~ 2 months: Recruitment of participants

3-5 months: experiment

6th month: result analysis

7~8 months: writing

16. References
