# Supplementary material for: Effects of a time-use intervention in isolated patients with coronavirus disease 2019: A randomized controlled study
Source: PLoS One. 2023 Jun 23;18(6):e0287118. doi: 10.1371/journal.pone.0287118 (PMC10289446; doi:10.1371/journal.pone.0287118)
Supplement: S3 File — (PDF) [file pone.0287118.s004.pdf]

심의용 연구계획서
(인간대상 연구용)

연구제목	시간 사용 중재가 격리된 경증 코로나 환자의 작업균형, 정신건강, 삶의 질에 미치는 영향			
연구자		성명	소속	직위	전화번호/E-mail
	연구책임자	박지혁	연세대학교	부교수	otscientist@yonsei.ac.kr
	공동연구자	정재휴	연세대학교	대학원 석사과정	jj1258@naver.com

1. 연구배경

COVID 19는 2019년 12월 중국 우한에서 시작되어 전 세계적으로 확산되었다. WHO는 COVID-19를 국제적 공중 보건 비상사태(PHEIC)로 선언하였으며 국내 또한 확진자가 급격히 늘어나며 장기화되는 국가긴급재난상황으로 COVID-19로 진단되거나 의심되는 환자를 격리하기 위해 엄격한 조치를 취하고 있다(WHO, 2020; 중앙재난안전대책본부, 2020).

COVID-19환자들 중 호흡부전, 패혈성 쇼크등의 중증이상의 신체적 증상을 보이는 환자는 19%이며 경한 증상을 보이는 환자는 81%로 중증환자보다 많다(Wu Z et al., 2020). COVID-19 환자들은 신체적 건강의 문제 뿐 아니라 긴 격리 기간, 감염 공포, 좌절, 지루함, 부적절한 정보, 재정적 손실, 낙인 등에 대한 스트레스 요인과 함께 불안 및 우울을 호소한다(Fan et al., 2020 ; Jiménez-Pavón, D et al., 2020). 2002년 중증 급성 호흡기 증후군(SARS)과 2012년 중동 호흡기 증후군(MERS) 생존자들이 퇴원 1년 후, 외상 후 스트레스장애, 우울증, 불안, 불면, 삶의 질 저하가 지속해서 관찰되는 모습을 통해 COVID-19 환자에게도 이러한 증상이 지속될 것으로 짐작할 수 있다(Lei et al., 2008 ; Stephen et al., 2020).

현재 국내의 COVID-19 양성 진단자들은 생활센터 및 전담병원 등으로 격리된다. 격리는 바이러스 축소에 큰 도움이 되며 감염을 막기 위한 최선의 선택이지만 격리에는 여러 가지 부정적인 면도 있다(Jiménez et al., 2020). 격리로 인해 개인의 가치관, 생활양식, 문화 등의 여러 요소가 포함되어 있는 복잡한 건강 척도인 라이프스타일에 변화가 생기며(Mandel, 1999; 김수미, 2016) 의미있는 작업의 균형을 파괴하기도 한다(Kamalakannan & Chakraborty, 2020). 무료함, 두려움, 불안, 외로움, 과도한 걱정, 우울 등의 스트레스를 경험하게 하여 신체적 건강과 심리적 질병의 발달 위험요소가 될 수 있다(Abad et al., 2010; Jiménez et al., 2020; Soheili, et al., 2020).

재난 피해자들에게 의미있는 작업의 참여는 스트레스 감소, 부정적인 반응 완화, 신체 및 정신적 회복을 촉진하여 개개인의 가치 있는 삶과 본래의 역할 복귀를 이끌어내는 중요한 역할을 할 수 있다(Yamkovenko, 2008; Smith & Scaffa, 2013; Kamalakannan & Chakraborty., 2020). 또한, 국가트라우마센터에서는 COVID-19환자들을 대상으로 낮 시간 동안 활동(운동, 활동, 규칙적 식사, 긍정적 태도 조언)을 하도록 권고하고 있다(국가트라우마센터, 2020). 따라서 라이프스타일이 변화되거나 방해되지 않고 활동적인 라이프스타일을 유지하여 작업의 균형을 유지하는 것이 건강에 중요할 것이다(Amin, K. P et al., 2020 ; Jiménez-Pavón, D et al., 2020).

작업균형은 일상활동의 조직화로 정의되는 말로 일, 노동, 가정관리, 육아, 여가 및 휴식활

동 등과 같은 다양한 참여를 허용하게 하여 건강과 웰빙을 가능하게 한다(Wilcock et al., 1997). 시간 사용은 사람들이 일상을 구성하고 구조화하는 방법의 기본이기 때문에 작업균형과 밀접한 관련이 있으며(Edgelow & Krupa, 2011) 사람들이 시간을 어떻게 보내는지 이해하는 것은 작업 균형 및 참여 연구에 특히 유용한 접근 방식이 될 수 있다(Pentland & McColl, 1999).

시간사용중재는 작업 영역 내에 시간을 적절하게 배분하여 작업 균형 상태를 유지하기 위한 중재로 인간작업모델(Model of Human Occupation; MOHO)에 이론적 근거를 둔다(Jeon, 2011). 척수손상환자, 뇌졸중환자, 정신과환자, 노인, 여성등 다양한 사람들을 대상으로 중재하여 이들의 우울 감소, 삶의 질 향상, 자아존중감, 생활만족도 등에 긍정적인 효과를 보였다(김영근, 2010; 김지훈, 2014; 박영주 등., 2015; 류성현 등., 2020 Edgelow & Krupa., 2011; Gutman et al., 2020).

COVID-19환자들의 경우 환경적, 심리적 요인으로 인해 라이프스타일의 변화, 작업의 불균, 정신건강의 문제가 보고되어 지지만 이에 대해 중재를 적용한 연구는 부족하다.

따라서 본 연구에서는 COVID-19 양성 진단 후 격리된 환자를 대상으로 작업적 시간사용 중재를 시행하여 환자들의 작업균형, 정신건강, 삶의 질에 미치는 영향을 분석하고자 한다.

2. 연구목적

본 연구에서는 COVID-19 양성진단을 받은 환자들을 대상으로 시간 사용 중재를 통해 환자들의 작업균형, 정신건강, 삶의 질에 미치는 영향에 대해 조사하고자 한다. 시간 사용 중재는 의미 있는 활동에 참여하는 시간을 증진하기 위해 일과를 스케쥴링 하는 것이다. 중재 전, 후에 작업균형, 정신적 건강 및 삶의 질을 측정하는 평가도구를 구글 온라인 설문 양식을 사용하여 진행한다. 본 연구결과는 COVID-19 양성진단자의 작업균형, 정신건강, 삶의 질에 도움이 되는 중재에 대한 기반이 될 것이며 환자들의 격리 경험 대처에 대한 임상적 근거 자료로 쓰일 것이다.

3. 연구 실시 기관명 및 주소

- 기관명 : 경기도의료원 안성병원
- 주소 : 경기 안성시 남파로 95

4. 연구 지원기관

없음

5. 연구기간

승인일로부터 8개월

6. 연구대상자

1. 선정기준
- COVID-19진단을 받고 격리되어 있는 자
- 의료진에 의해 경증으로 분류 된 자
  • 호흡기계 기저질환이 없는 자
  • 산소포화도 95%이상 측정되며 혈역학적으로 안정된 자
  • 흉부촬영영상에서 저명한 폐렴소견이 관찰되지 않는 자

- 만 18세 이상인 성인
- 연구 목적을 이해하고 연구 참여에 자발적으로 동의한 자

2. 배제기준
- 의학적으로 안정되지 않은 자
- 한국어 사용이 어렵게나 의사소통에 문제가 있는 자

7. 예상 연구대상자 수와 산출 근거

본 연구는 선행연구 분석을 통하여 예상 연구 대상자 수는 총 50명으로 산출하였음

* 선행연구를 통한 연구대상자 선정
- COVID-19환자를 대상으로 시행한 이완활동이 불안, 수면의 질에 미치는 영향에 대한 연구
Liu, K et al., 2020 - COVID환자 51명

- 정신과 증상을 보이는 환자를 대상으로 작업치료(면담, 활동기반중재)를 시행한 연구
Hoshii et al., 2013 - 정신과 증상을 보이는 환자 59명
Shimada et al., 2016 - 정신과 환자 51명

8. 연구대상자 모집

1. 경기도의료원 안성병원에 입원한 환자들을 대상으로 전화를 통해 연구에 대한 충분한 안내를 시행한 후 연구 참여에 대한 동의를 구하여 참여하게 함
2. 개인의 사생활 보호 및 비밀유지
- 본 연구의 참여로 대상자에게서 수집되는 개인정보는 연구 종료 후 1년까지 사용되며 수집된 정보는 개인정보보호법에 따라 적절히 관리될 예정임
- 해당 개인정보는 연구자 이외에는 제공되지 않으며. 연구 종료 후 개인정보는 연구종료 후 3년간 보관되며 이후 폐기될 예정임
- 대상자의 개인정보는 기밀이 유지되도록 관리하며, 신원을 파악할 수 있는 기록은 관리번호를 부여하여 익명화 하고, 연구관련 자료는 연구 관련자 외 접근을 금하고 시건 장치가 있는 곳에 보관할 예정임
- 또한 연구 결과가 출판될 경우에도 대상자의 신원은 기밀이 유지될 것임

9. 연구대상자 동의

- 연구자는 대상자에게 연구 목적과 배경 등을 설명하고 동의서를 보여준 후 연구 참여자의 동의를 얻음
- 연구 참여자는 참여 도중 언제든지 동의를 철회할 수 있으며, 이로 인해 불이익은 없음

10. 연구방법

1. 연구 대상자 대정
- 연구 대상자 50명을 실험군 25명, 대조군 25명으로 무작위 배정함
- 선택편향을 일으키지 않기 위해서 블록의 크기에 무작위배정을 사용함(강현., 2017). 50명의 피험자를 A, B 두 개의 군에 배정비 1 : 1인 블록 무작위 배정 시행. 본 연구에서는 50명의 sample size를 블록 크기는 6, 8, 10, 12, 14를 사용하였다. Excel spread sheet를

사용하였으며 상세 과정은 이하와 같다.
- 먼저 빈 Excel 스프레드시트를 열고, 셀 A1, B1, C1, D1, E1에 머리글로 Sequence, Random1, Random 2, Block, Group을 각각 입력한다. Sequence 열인 셀 A2에서 A51까지에는 1부터 50까지의 수를 순서대로 입력하고, Block 열인 셀 D2에서 D51까지에는 6을 6개, 8을 8개, 10을 10개, 12를 12개 순서대로 입력한다. 여기에서 각 숫자는 블록의 크기를 의미한다. 다음으로 Group 열인 셀 E2에서 E21에는 블록 크기의 절반에 해당하는 수의 A와 B를 순서대로 입력한다. 다음으로 각 블록의 시작인 셀 B2, B8, B16, B26, B38에 함수 = rand()을 입력한다. 함수 = rand()로 인해 스프레드시트가 업데이트될 때마다 난수가 바뀔 수 있으므로, 선택하여 붙여넣기-값을 이용하여 복사한다. 그렇게 되면 B 열에 있는 함수는 숫자로 변환되어 업데이트될 때마다 바뀌지 않는다. 셀 B2, B4, B8, B14의 오른쪽 하단의 모서리를 클릭하여 각 셀 아래 셀에 복사하여 넣는다. 다음으로 머리글 Random2 아래 20개의 셀에 = rand()을 입력한다. 다음으로 머리글 Random1부터 Group까지 드래그하여 선택하고, 홈-정렬 및 필터-필터를 선택한다. 다음으로 Random2 오름차순 정렬, Random1 오름차순 정렬을 시행하면 블록 크기의 무작위 배정을 동반한 블록 무작위 배정이 시행되었음을 확인할 수 있다.

2. 중재 적용
* 연구 대상자들에게 적용되는 모든 중재는 본인의 병실에서(병실 밖으로의 환자의 이동은 없음)수행함
1) 시간사용 중재(스케쥴링)
- 실험군에게 시행
- 총 10회기로 2주간, 주 5회(평일 매일)실시
- 첫날은 Step1~3를 시행(약 40분 소요)
- 둘째 날부터 마지막 날 까지는 Step4를 시행(약 15분 소요)
- 중재는 전화로 진행되며 중재에 필요한 도구는 치료사가 병실 앞에 두면 환자가 가져가는 방식으로 제공(현재 치료사가 병실 방역업무로 인해 매일 병동 출입 중으로 이에 대한 추가적인 문제는 없음)
- 절차

Step	Program	Contents	Evidence
1	시간사용 분석	- K-LBI결과를 통해 작업의 불균형을 확인<br>- 작업설문지(Occupational Questionnaire)를 통해 시간사용량을 분석	Smith et al.(1986)<br>김영근(2010)<br>김지훈(2014)
2	작업 선정	- 입원 후 시간이 고정된 일과 생각하기 : 식사 나오는 시간, 활력징후 측정 시간 등<br>- 해야 하는 기본적 활동 나열하기 : 수면, 개인위생 등, 학생일 경우 원격 수업 듣는 시간, 재택근무가 가능할 경우 직장인일 경우 일하는 시간 등<br>- 병실에서 가능한 활동 목록을 제공하여 치료사와 면담을 통해 추가적으로 하고 싶은 활동 선정<br>: 스트레칭, 고무밴드(Thera-band)와 아령을 사용한 신체활동, 이완활동을 통한 스트레스관리, 수공예	홍승표 등 (2008)<br>Law et al(1998)<br>김지훈(2014)을 기반으로 재구성 함

		활동(뜨개질, 컬러링북, 스크래치북, 포일아트, 보석자수, 드림캐쳐만들기 등) 등	
3	활동배치	- 작업설문지(OQ)를 바탕으로 무의미한 시간대에 2단계에서 선정한 의미 있는 작업을 배치함<br>•고정된, 기본적 활동들의 시간 정하기<br>: 일어나는 시간, 잠자는 시간, 개인위생 시간 등<br>•하고 싶은 활동들의 시간 정하기<br>: 신체활동 시간, 여가활동 시간 등	김영근(2010)<br>김지훈(2014)
4	실천하기 및 연구자 개입	- 계획대로 시행하기<br>- 연구자와 매일 아침 전화하여 전날의 시간사용에 대해 면담하여 활동을 수정 보완 함	김영근(2010)<br>박수진(2015)

2) 자가교육 및 운동을 위한 교육자료 제공
- 대조군에게 시행
- 입원 시 1회 제공, 30분 실시
- 교육자료는 입원 시 필요한 서류와 함께 제공 되며 교육은 전화로 시행됨
: 교육자료는 코로나 환자들을 대상으로 제작되었으며 관련기관에 문의하여 ‘출처 표기 시 교육자료 사용 및 논문게재에 문제없음’을 확인하였음
: 교육자료는 스트레스 관리 및 안정화기법과 관련되어 있음

(국가트라우마센터)

3) 의학적 처치 및 보존적 치료
: COVID-19 대응지침에 따른 격리 및 보존적 치료
: 의료진에 의해 실험군과 대조군에게 동일하게 시행됨

3. 연구 절차

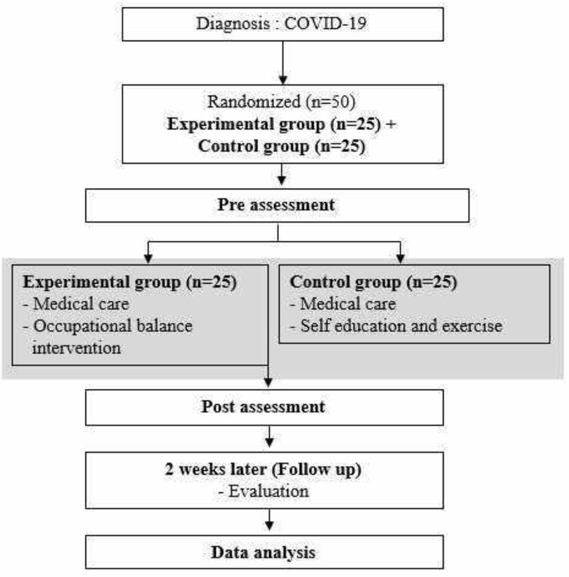

11. 관찰 항목

1. 연구 참여자의 일반적 정보
- 의무기록정보를 통해 성별, 연령, 발병기간 등의 정보를 수집함
문자로 전송 가능한 구글설문지 형식을 이용한 다음의 자가보고식 평가도구를 사용하여 작업균형, 정신건강, 삶의질에 대하여 평가
- 사전(첫째 날), 사후(중재가 마무리되는 10일째), 후속평가(퇴원 후 2주후)를 시행함

2. 작업 균형 : K-LBI(Korean Version of Life Balance Inventory)
작업 균형을 측정하는 평가도구로 Matuska(2012)가 라이프밸런스모델을 기반으로 개발된 평가도구를 박상미 & 박지혁(2019)이 한국어로 번안하여 표준화하였다. 53개 활동(ADL, IADL, 일, 놀이, 휴식과 수면, 여가, 교육, 사회참여)과 4개 하위항목(건강, 아이덴티티, 관계, 도전)으로 구성되어 있으며 1점(항상 원하는 시간보다 적게 혹은 많게)에서 3점(대체로 내가 원하는 만큼)까지 5개 척도로 응답한다. 3점에 가까울수록 작업 간에 균형이 잘 잡혀있다고 해석되어진다. Cronbach’s α는 노인 0.88, 성인 0.83으로 나타났다(박상미 & 박지혁, 2019).

3. 정신건강
 1)우울 : PHQ-9 : (Patient Health Questionnaire-9)
우울증의 중등도를 진단, 선별, 모니터링 및 측정하는데 사용되는 자가보고식 도구로 Spitzer 등(1999)이 개발한 평가도구를 최홍석 등(2007)이 번안하여 표준화하였다. 총 9문항으로 구성되어 있으며 4점척도를 사용하여 ‘없음’, ‘2~3일’, ‘7일이상’, ‘거의매일’로 평가한다(최홍석 등., 2007). Cronbach α는 0.92로 나타났다(박승진 등., 2010).

2)불안 : SAS(self rating anxiety scale)
William W. K. Zung이 1971년 개발한 평가도구로 불안 관련 증상이 있는 환자의 불안수준을 측정하는 도구임. 20개의 질문 중 15개의 불안 수준 증가 질문과 5개의 불안 감소 질문으로

구성되어 있으며 4점척도를 사용하여 '거의 없음' 에서 '거의 매일' 까지로 구성되어 있음.

3) 수면의 질 : Korean version of Insomnia severity index(ISI-K)
ISI-K는 Charles등에 의해 불면증의 야간 및 주간 구성 요소의 심각도를 평가하기 위해 고안된 도구로 2014년 조용원에 의해 번안되었다. 총 7개의 항목으로 구성되어 있으며 5점 리커트 척도를 사용한다. 7점 이하는 정상, 8-14점은 역치 이하 불면증, 15-21점은 중등도 불면증, 22-28점 심각한 불면증으로 해석된다. Cronbach α는 0.92로 나타났다(Cho et al., 2014).

4) 지루함 : Multidimensional State Boredom Scale-8(MSBS-8)
참여자들의 지루함을 평가하기 위한 도구로 2011년 Fahlman 등에 의해 고안된 29항목의 MSBS를 8항목으로 축약하여 개발한 것이다. 7점 리커트 척도를 사용하며 높은 점수는 더 큰 지루함을 반영한다(Hunter et al., 2015).

5) COVID-19공포 : FCV-19S(Fear of COVID-19 scale)
COVID-19에 대한 두려운을 측정하는 자가보고식 평가도구로 두려움의 감정적 반응과 증상적 표현의 영역에 해당하는 7문항의 항목으로 구성된다. 5점척도를 사용하며 점수가 높을수록 COVID-19에 두려움이 높은 것이다. Cronbach α는 0.82로 나타났다(Ahorsu et al., 2020).

4. 삶의 질 : Korean version of WHOQOL-BREF
(World Health Organization Quality of Life Assessment Instrument-BRIEF)
건강관련 삶의 질을 평가하기 위한 도구로 1998년 WHOQOL Group에 의해 개발된 WHOQOL-BREF를 2000년 한국형으로 번안한 것으로 총 26문항으로 구성되어 있으며 각 문항은 1~5점 리커트 척도를 사용한다. 전체적인 건강관련 삶의 질과 함께 4가지 하위 영역인 신체적 건강 영역, 심리적 건강 영역, 사회적 관계 영역, 환경 영역으로 이루어져 있다(Whoqol Group, 1998). Cronbach's α는 0.898, 검사-재검사간 신뢰도는 0.436~0.731범위로 나타났다(민성길, 이창일, 김광일, 서신영, & 김동기, 2000).

12. 효과 평가 기준 및 방법

- 중재 전, 후, 퇴원 후 작업균형, 정신건강, 삶의질의 변화를 확인하고 통계적으로 분석 할 때 유의수준 0.05를 기준으로 하여, p 값이 0.05 이하를 효과의 기준으로 함

13. 안전성 평가 기준 및 평가 방법

- 본 연구는 근거가 충분하게 있는 중재이며 평가 및 중재가 치료사와 1:1로 이루어지기 때문에 안전 관리가 가능함
- 본 연구와는 별개로 간호실에서 COVID-19 환자들의 vital sign은 주기적으로 측정 및 CCTV로 환자의 상태를 지속적으로 파악하고 있기 때문에 만일, 평가, 중재 중 혹은 후에 환자가 피로도 혹은 불편감을 호소한다면 즉각적인 의학적 대처가 가능함

14. 자료분석과 통계적 방법

- Descriptive analysis : 대상자의 일반적 특성
- 각 평가의 사전 - 사후 평가, 사후 - 후속 평가 평균점수를 전 - 후 비교하여 효과를 분석
  : Paired t-test 또는 Wilcoxon signed-rank test를 사용하여 동일 표본에서의 전-후 차

이를 분석
: Indipendent t-test 또는 Mann-Whitney u test를 사용하여 실험군과 대조군의 차이를 분석
- 각 평가의 사전 - 사후 - 후속 평가 평균점수를 비교하여 효과를 분석
: ANOVA 또는 Kruskal-Wallis test를 사용
- 자료분석을 위해 spss 25.0를 사용할 예정

15. 연구수행 일정표

* IRB 승인일 이후 ~ 2개월 : 대상자 모집 및 선정
3~5개월차 : 실험
6개월차 : 결과분석
7~8개월차 : 논문작성

16. 참고문헌

Abad C, Fearday A, Safdar N. Adverse effects of isolation in hospitalised patients: asystematic review. J Hosp Infect 2010; 76: 97-102 [PMID: 20619929 DOI:10.1016/j.jhin.2010.04.027]

Amin, K. P., Griffiths, M. D., & Dsouza, D. D. (2020). Online Gaming During the COVID-19 Pandemic in India: Strategies for Work-Life Balance. International Journal of Mental Health and Addiction, 1-7.

Edgelow, M., & Krupa, T. (2011). Randomized controlled pilot study of an occupational time-use intervention for people with serious mental illness. American Journal of Occupational Therapy, 65(3), 267-276.

Fan, P. E. M., Aloweni, F., Lim, S. H., Ang, S. Y., Perera, K., Quek, A. H., ... & Ayre,T. C. (2020). Needs and concerns of patients in isolation care units-learnings from COVID-19: A reflection. World Journal of Clinical Cases, 8(10), 1763.

Gutman, S. A., Balasubramanian, S., Herzog, M., Kim, E., Swirnow, H., Retig, Y., & Wolff, S. (2020). Effectiveness of a tailored intervention for women with attention deficit hyperactivity disorder (ADHD) and ADHD symptoms: A randomized controlled study. American Journal of Occupational Therapy, 74(1), 7401205010p1-7401205010p11.

Jeon, B. J. (2011). A study on factors influencing elderly's life time use and occupational balance (Doctoral dissertation). Sungkyunkwan University, Seoul. Jiménez-Pavón, D., Carbonell-Baeza, A., & Lavie, C. J. (2020). Physical exercise as therapy to fight against the mental and physical consequences of COVID-19 quarantine: Special focus in older people. Progress in cardiovascular diseases.

Kamalakannan, S., & Chakraborty, S. (2020). Occupational therapy: The key to unlocking locked-up occupations during the COVID-19 pandemic. Wellcome Open Research, 5.

Law, M., Baptiste, S., McColl, M., Opzoomer, A., Polatajko, H., & Pollock, N. (1990). The canadian occupational performance measure: An outcome measure for occupational therapy. Canadian Journal of Occupational Therapy, 57(2), 82-87. doi:10.1177/000841749005700207

Lei, B. L., Zhou, Y., Zhu, Y., Huang, X. Y., Han, S. R., Ma, Q., . . . Li, Y. Q.(2008).

Emergency response and medical rescue in the worst hit Mianyang areas after the Wenchuan earthquake. Journal of Evidence-Based Medicine, 1(1), 27-36.

Pentland, W. E., & McColl, M. A. (2002). Application of time use research to the study of life with a disability. In Time use research in the social sciences (pp. 169-188). Springer, Boston, MA.

Smith, N. R., Kielhofner, G., & Watts, J. H. (1986). The relationship between volition, activity pattern and life satisfaction in the elderly. American Journal of Occupational Therapy, 40(4), 278-283. doi:10.5014/ajot.40.4.278

Smith, T. M., & Scaffa, M. E. (2013). Providing occupational therapy for disaster survivors. In B. A. Schell, G. Gillen, M. Scaffa, & E. S. Cohn (Eds.), Willard and Spackman's occupational therapy (pp.962-971). Baltimore: Lippincott Williams & Wilkins.

Wilcock, A. A., Chelin, M., Hall, M., Hamley, N., Morrison, B., Scrivener, L., ... & Treen, K. (1997). The relationship between occupational balance and health: A pilot study. Occupational Therapy International, 4(1), 17-30.

Wu, Z., & McGoogan, J. M. (2020). Characteristics of and important lessons from the coronavirus disease 2019 (COVID-19) outbreak in China: summary of a report of 72 314 cases from the Chinese Center for Disease Control and Prevention. Jama, 323(13), 1239-1242.

Yamkovenko, S. (2008). Occupational therapy's role in disaster relief. Retrieved from http://www.aota.org/about-occupational therapy/professionals/mh/articles/disaster-relief.aspx

강현. (2017). 무작위 배정과 배정확률에 변화를 주는 무작위 배정. Anesthesia and Pain Medicine, 12(3), 201-212.

김수미. (2016). 1 인 가구의 라이프스타일과 노후준비. 전남대학교, 광주. Retrieved from http://www.riss.kr/link?id=T14208855 (국내박사학위논문)

김영근. (2010). 장애노인을 위한 효율적인 시간사용중재 효과. 재활복지, 14, 205-224.

김지훈. (2014). 효율적인 시간사용 중재가 척수손상 환자의 우울과 삶의 질에 미치는 영향-단일 사례 연구. 대한인지재활학회지, 3(1), 59-74.

류성현, 박수진, 김민혁, & 황도연. (2020). 시간사용중재가 뇌졸중 환자의 생활만족도 및 자아존중감에 미치는 영향. 재활치료과학, 9(3), 91-102.

박영주, 김유석, 박수정, 방지현, 송지원, & 장문영. (2015). 시간관리 프로그램의 적용이 문제음주 대학생들의 음주습관과 자기효능감에 미치는 영향. 대한감각통합치료학회지, 13(1), 57-66.

홍승표, 이지은, 박선희. (2008). 재활병원에 입원한 척수손상환자들의 작업수행 문제들과 시간사용에 관한 연구. 대한작업치료학회지, 16(4), 89-98.

17. 연구책임자 경력사항 (별첨 1. 참고)
18. 연구비 소요내역서(과제연구의 경우 해당) (별첨 2. 참고)

별첨 1.

연구책임자 경력사항

(1) 인적사항

<table>
<tr><td>성 명</td><td colspan="6">박지혁</td></tr>
<tr><td rowspan="5">직 장</td><td>기관명</td><td colspan="3">연세대학교</td><td>전 화</td><td>033-760-2450</td></tr>
<tr><td>전 공</td><td colspan="3">작업치료학과</td><td>F A X</td><td>033-760-2496</td></tr>
<tr><td>부 서</td><td>작업치료학과</td><td>직위</td><td>부교수</td><td>휴대전화</td><td>010-9304-9706</td></tr>
<tr><td rowspan="2">주 소</td><td>우편번호</td><td colspan="2">220-710</td><td rowspan="2">E-mail</td><td rowspan="2">otscientist@yonsei.ac.kr</td></tr>
<tr><td colspan="3">강원도 원주시 흥업면 연세길 1
연세대학교 원주캠퍼스 (백운관 132호)</td></tr>
</table>

(2) 주요 경력

연 도		근무기관	직위(직명)	비 고
부터	까지			
1995. 3 ~ 2003. 2	연세대학교	작업치료학	학사	
2003. 3 ~ 2005. 2	연세대학교	작업치료학	석사	
2005. 3 ~ 2010. 2	University of Kansas Medical Center	Therapeutic Science	박사	
2011.03.01. ~ 현재	연세대학교 작업치료학과	부교수		

(3) 주요 연구실적 (지난 3년간) 및 논문 발표실적

논문명	게재일	서지정보(게재 저널명, 권(호), 페이지)
지역사회 거주 고령자의 라이프스타일 측정도구에 관한 조사: 경향과 활용전망.	2019.08	재활치료과학, 8(3), 7-29
건강과 삶의 질에 영향을 미치는 지역사회 고령자의 라이프스타일 구성요소에 관한 사전연구: 델파이 조사.	2019.09	대한작업치료학회지, 27(3), 105-120.
완곡추적 안구운동과 경부신전근 진동자극법의 결합중재가 만성 뇌졸중환자의 편측무시와 일상생활활동 수행에 미치는 영향: 단일대상 연구	2019.03	International Journal of Oral Biology, 44(1), 71-83
로봇치료가 파킨슨병 환자의 상지 기능에 미치는 영향	2018.08	신경재활치료과학, 7(3), 59-78
Does cognition-specific computer training have better clinical outcomes than non-specific computer training? A single-blind, randomized controlled trial	2018.02	Clinical rehabilitation, 32(2), 213-222
오차배제학습과 시간차회상을 이용한 작업기반 훈련이 경도 혈관성 치매환자의 과제 수행능력과 만족도에 미치는 영향: 개별실험 연구	2018.02	신경재활치료과학, 7(1), 51-62
Development of an Item List to Assess Bilateral Upper Extremity Function of Stroke Patients with Hemiplegia	2018.01	Neurorehabilitation, 41(1), 37-42
작업균형의 개념과 측정에 관한 고찰: 경향과 치료적 활용전망	2017.08	한국웰니스학회지, 12(3), 115-125
Does cognition-specific computer training have better clinical outcomes than non-specific computer training? A single-blind, randomized controlled trial	2017.07	Clinical Rehabilitation, 32(2), 1-10
Validity and Reliability of the Korean Version of the Utrecht Scale for Evaluation of Rehabilitation-Participation	2017.01	Occupational Therapy International, 2017, 5pages
