# Supplementary material for: Effects of a time-use intervention in isolated patients with coronavirus disease 2019: A randomized controlled study
Source: PLoS One. 2023 Jun 23;18(6):e0287118. doi: 10.1371/journal.pone.0287118 (PMC10289446; doi:10.1371/journal.pone.0287118)
Supplement: Attachment — Submitted filename: Response to reviewers.docx [file pone.0287118.s005.docx]

**PLOS ONE - REVISION**

**COMMENTS TO THE AUTHOR:**

Reviewer #1: The study aims to investigate the effect of time-use intervention on occupational balance, mental health, and quality of life in COVID-19 patients who were forced to isolate.

**-** **I appreciate for your kind review.**

Abstract

1. The method section in the abstract requires revision by describing it in a paragraph and not structuring it. Education for self-activity to be mentioned for the control group. Ensure what is written in this section is similar or applied to other section(s) or Figure(s).

**Answer)** As your recommendation, method section in the abstract has been rewritten. Also, we have added the contents of self-activity education implemented for the control group. Also, we mentioned time-use intervention and education for self-activity in section of intervention in abstract.

**→** **Methods:** Randomized controlled single center clinical trial. Forty-one patients (19 in the experimental group, 22 in the control group) with COVID-19 were recruited from February 1 to March 19, 2021. Participants were randomly assigned to receive time-use intervention, or the education for self-activity. Time-use intervention is to plan a daily routine to engage in meaningful occupations. It consists of 4 steps: time use analysis, occupation selection, arrangement of activities, and practice and occupational therapist intervention. The control group is educated for self-activity and spends time autonomously. (Page 2, Line 36-42)

Methods

Time use intervention

2. Line 96, information on allocation concealment and blinding is to be provided. The person who assigned the subjects to intervention and control group to be stated.

**Answer)** It was a non-blinded trial because the intervention in this study was not easily blinded. For the allocation concealment, a doctor, not a researcher, performed block randomization.

**→** It is a non-blinded trial, and the participants were assigned in the order of the beds by the physician after he had checked medical state. (Page 5, Line 101-102)

3. Line 106-107, Figure 2 Step 4, for the statement ‘Provide the supplies for occupational performance’, more details are to be provided.

**Answer)** Ambiguous sentences were corrected. Supplies are provided for the occupation selected in step 2 by occupational therapist. Materials are different with patients’ selection.

→ The participant performed occupations on time. (Page 6, Line 122) The occupational therapist provided materials which were selected in step 2 (punching embroidery, scratch art etc.) once a day. Materials are different with patients’ selection. (Page 6, Line 126-129)

4. 116, standard care to be defined.

**Answer)** As your comment, we defined standard care.

→ Both the experimental and the control group were provided symptomatic treatment for COVID-19-related symptoms, and antiviral therapy. (Page 8, Line 141-143)

Secondary outcome measures

5. Line 132 -137, for those questionnaires that were not stated in Korean version, it is not clear whether the participants answered the questionnaires via a mix of English and Korean versions questionnaires. The mode of administration whether self-administered or interviewed to be clearly stated.

**Answer)** For better understanding, we had cited English version of outcome measures. As your question, we have clarified that the resulting measurement used the Korean version.

**→** Except for MSBS-8, outcome measures were used be Korean version. The MSBS-8 was translated into Korean by research team. All outcome measurements were performed by the participants themselves with questionnaires in Google Form. (Page 9, Line 165-167)

Statistical analysis

6. Line 142, For Fisher’s exact test, one or two-tailed test to be stated.

**Answer)** Two-tailed fisher's exact test was used so we changed the word ‘fisher's exact test’ to ‘two tailed fisher's exact test’.

→ For the comparison of the demographic and clinical characteristics between the two groups, the independent t-test, the chi-square test, and two-tailed fisher's exact test was used.

(Page 9, Line 171-175)

7. Line 150, for the post-hoc power analysis, more detail is to be provided on how the power is derived when involving two intervention groups.

**Answer)** Added information used for post-hoc power analysis.

**→** Post-hoc power analysis was conducted to examine the statistical power of the group and time differences (Cohen’s d = 1.21, α error = .05, Sample size group 1 = 19, sample size group 2 = 22). (Page 9, Line 183-185)

8. The reason the sample size calculation was not performed in the study is to be stated.

**Answer)** The sample size was established based on previous studies of similar interventions. To supplement this, post-hoc power analysis was performed.

→ The sample size was established based on previous studies of similar interventions. Post-hoc power analysis was conducted to examine the statistical power of the group and time differences (Cohen’s d = 1.21, α error = .05, Sample size group 1 = 19, sample size group 2 = 22). (Page 9, Line 182-185)

Results

9. Line 183-184, 188-189, 196, 205-208, 217 etc some p values were different from Table 2. Also check if p= .001 is the actual value or requires symbol <. Since the p values are available in the table there is no need to use footnote Line 242. Actual p-value to be used in the text even though they are not statistically significant except when summarizing all the p values. This also applies to the text results describing Figure 3 and other sections in the manuscript.

**Answer)** The p values written as p<.05 and p<.001 in the manuscript and figure3 have been corrected to the actual values. (Page 12, Line 217. 218. 220. 222. 223. 23. 234. 235. 239. 240. 242-244. 258) Also, Footnote of table 2 in Line 242 (*p<.05, **p<.001) was erased. (Page 27)

10. Line 190, the sentence ‘The change in the control group was not statistically significant (p > .05)’ to be revised to ‘The change in the control group for all the occupational balance items was not statistically significant (ps > .05)’

**Answer)** As your recommendation, it was revised.

→ The change in the control group for all the occupational balance items was not statistically significant (p=.05). (Page 12, Line 225-226)

11. Line 207, 209, the sentence requires revision,

**Answer)** As your recommendation, we revised the sentence.

→ By contrast, PHQ-9 scores (p=.032), ISI-K scores (p=.005), MSBS-8 scores (p<.0001), and FCV-19S scores (p=.031) were increased in the control group. Also, SAS decreased in the experimental group and increased in the control group. However, the difference was not statistically significant in both groups (p>.05). (Page 13, Line 249-251)

12. Table 1, the statistical tests to be denoted in table footnote. Having said that, based on CONSORT statement, all statistical tests performed at baseline are to be avoided. Since the symbol % is highlighted after the variable name, all individual symbol % for the figures to be omitted.

**Answer)** The independent t test, chi-square, and Fisher's extract test were performed for descriptive data analysis. As your comment, we added on footnote of table 1 about statistical test. According to the CONSORT statement, table 1 has been modified with values that have not been tested for significance (p value of duration of isolation: .62 to .84). Also, % was deleted. (Page 25)

13. Table 2, effect size index and 95% CI could be presented.

**Answer)** We added effect size index and 95% CI on Table 2. We calculated cohen`s d within group and partial omega squared in interaction. (Page 26-27)

14. Line 242, p<.00 to be revised.

**Answer)** As your first comment in the result, footnote of table 2 in Line 242 (*p<.05, **p<.001) was erased. (Page 27)

15. Figure 1, the intervention that the experimental and control group received to be placed in the Figure. The assessment time point/period is to be stated at follow-up. The allocation number for the intervention and control group for those who did not meet the criteria are to be separated and placed in another box. This is to separate the number of patients who received the interventions.

**Answer)** The assessment period was stated at follow-up in Figure 1. However, it is difficult to write an accurate time because the hospitalization period is different for each patient depending on the medical condition. We changed "post assessment" to "post assessment at discharge". Also, descriptions of patients who discontinued during the experiment were separated and placed in another box.

<Part of Figure 1>

16. In Figure 2 footnote, to denote both groups received standard care.

**Answer)** Added footnote in Figure 2; Both groups received standard care.

<Part of Figure 2>

Discussion

17. Line 228, decimal point to be standardised.

**Answer)** It has been standardized to describe up to 3 decimal places. W replaced 0.37 with .378. (Page 11, Line 242)

→ However, in the control group, the mean of the total K-LBI score decreased from 2.05±0.40 to 1.95±0.3 (p=.378). (Page 14, Line 268-269)

18. 268, for the statement small sample size, it requires justification e,g. sample size calculation.

**Answer)** We wrote that because the absolute number of samples was small (experimental group, n =19). However, it was justified through post-hoc analysis (Page 12, Line 227-228) and effect size of each outcome measures (Page 26-27).

→ However, it was justified through post-hoc analysis and effect size of each outcome measures. (Page 19, Line 311-312)

References

19. List of references did not conform to the journal format.

**Answer)** We modified journal format suitable for Plos one's guidelines.
