# Supplementary material for: Effects of a time-use intervention in isolated patients with coronavirus disease 2019: A randomized controlled study
Source: PLoS One. 2023 Jun 23;18(6):e0287118. doi: 10.1371/journal.pone.0287118 (PMC10289446; doi:10.1371/journal.pone.0287118)
Supplement: Attachment — Submitted filename: Comments.docx [file pone.0287118.s006.docx]

Effects of Time-use Intervention in Isolated Patients with coronavirus disease 2019: A Randomized Controlled Study

The authors evaluated a new protocol for time-use among patients suffering from infectious diesases requiring isolation which is very important in this current situation when the world is fighting for prevention and control of the ongoing COVID-19 pandemic. If enough evidence is generated through the intervention trials for improving mental health, occupational balance and quality of life during such crisis where patients require to stay isolated for long period of time from family and friend, it can be impactful for improving patient’s compliance to treatment even during convalescent period.

The manuscript may be considered for publication after addressing the following comments.

Methods:

Line 79: What assumptions were made for including 50 patients for this trial? Justification for this sample size need to be addressed. To study effectiveness of a new intervention protocol, a considerable number of samples needs to be included to provide a significant result of the analysis.

Line 80: it is not clear if the patients were isolated in single cabin or ward. How were the study participants followed-up?

Line 83: What measures taken to avoid bias? If a patient assigned to control group gets into contact with intervention group, there is a risk that control group participants may adapt some of the tasks assigned to intervention group. Hence was any measure taken to avoid the introduction of this bias?

Line 97: How were the participants instructed or briefed about the intervention protocol. Was there any formal training provided to the participants in the intervention arm on the procedures to comply the intervention protocol?

Line 101: How is the occupation selection done? Please define the steps to clarify.

Results

Line 207: Delete “was” as it is abundant in this sentence.

Table 2- The statistical tests used in this analysis should be mentioned in the footnote with the appropriate level of significance.

Discussion:

Line 229: Authors can emphasize on the scoring range found from previous studies that correlates with the level of stress.

Line 261-266: Elaborate the discussion on how the QOL scores differ in different settings of isolation. For example, isolating in a single cabin, ward, or institute with having or not having activities involved during the isolation period.

Conclusion:

Line 291-294: Authors should emphasize the prospective impact of this new developed protocol of the time-use intervention as well as way forward in the field of mental health and challenges.
