# Supplementary material for: Effects of a time-use intervention in isolated patients with coronavirus disease 2019: A randomized controlled study
Source: PLoS One. 2023 Jun 23;18(6):e0287118. doi: 10.1371/journal.pone.0287118 (PMC10289446; doi:10.1371/journal.pone.0287118)
Supplement: Attachment — Submitted filename: Response to Reviewers.docx [file pone.0287118.s007.docx]

Review Comments to the Author

Please use the space provided to explain your answers to the questions above. You may also include additional comments for the author, including concerns about dual publication, research ethics, or publication ethics. (Please upload your review as an attachment if it exceeds 20,000 characters)

**Reviewer #1:**

1. Table 1 footnote, typo error ' Fisher's extract test'.

**Answer>** As your recommendation, we modified 'Fisher's extract test' to 'fisher's exact test. Thanks for finding the typo error.

- Independent t test, Chi-square, and two-tailed fisher's exact test' were performed (Page 22, Line 519)

**Reviewer #2:**

1. One more thorough review of the English language editing is required.

**Answer>** As your recommendation, I reviewed the English language edit again.

2. Please clarify the primary objective and secondary objective in the objective part of the abstract.

**Answer>** As your recommendation, we clarified the primary objective and secondary objective in the objective part of the abstract. The primary objective is to find out the impact of time use intervention on the occupational balance of COVID-19 patients, and the secondary objective is the impact on mental health and quality of life.

**-** The primary objective of this study was to determine the effectiveness of time-use intervention on occupational balance in isolated COVID-19 patients. The impact on secondary outcomes including mental health and quality of life were also assessed. (Page 2, Line 26-29)

3. Please slightly describe the **occupation imbalance/balance and time use intervention** at its first use in the introduction. It is not clear while reading the introduction.

**Answer>** As your recommendation, we added a brief explanation of occupation imbalance/balance and time-use intervention in the introduction and rearranged the introduction to make it easier to understand.

- Occupation balance is defined as the organization ….. occupation area to maintain occupation balance [8, 9] (Page 4, Line 71-82)

4. How was the informed consent of the patient taken if the written form were waived? Write about the approval of the hospital to undertake the study.

**Answer>** As your comment, we added an explanation for obtaining informed consent from the patient in method section. I have attached the Google form that I actually used to obtain consent below to help your understanding. The green section is research information and contents. If patients had voluntary participated in the study, they pressed the "I agree" button in red section. Also, we conducted the study in compliance with the hospital's ethical regulations.

- As an alternative to written consent, it was designed to proceed with the pre-evaluation when online consent was given by clicking the “I agree” button on the Google form with tablet PC.. (Page 6, Line 124-126)

5. Describe a little bit about the study setting. What is the duration of the patient staying at the hospital? On what day of residing was the intervention taken, and how long and how many times was the intervention given is not clear in detail? It is unclear about the duration between pre-and post-assessment. Provide detailed information on the time length taken in each step from the start of enrolment to final study completion.

**Answer>** The isolation period (hospitalization period) of the patients varied from 11 to 24 days depending on the patient's condition. Inconsistent length of hospital stay may affect the results, but there was no significant difference in the duration of isolation between the two groups as shown in Table 1. Time-use intervention was performed over 7 days depending on hospitalization period in the experimental group. Education for self-activity is provided once and allows the patient to act autonomously during hospitalization. Both interventions are premised on the patient's activity during hospitalization. Also, evaluations were conducted once each after admission and before discharge for both groups. Therefore, duration between pre-and post-assessment is different for each patient. Duration between pre-and post-assessment is similar with the hospitalization period. Finally, Steps 1 to 3 of time-use intervention were performed on the first day of intervention. As your comment, we added that contents to the manuscript.

- The experimental group received time-use intervention and the control group received education for self-activity. Interventions were applied every day during the isolation period. All participants received the intervention individually. (Page 7, Line 146-148)

- All outcome measurements were written in a google form and performed by the participants themselves with google form of table PC. Evaluations were conducted after admission and before discharge for both groups. (Page 11, Line 237-239)

- The isolation period of the patients varied from 11 to 24 days depending on the patient's condition. (Page 13, Line 271-272)

- Education for self-activity was conducted once in the control group. (Page 8, Line 169)

- Time-use intervention was performed over 7 days depending on length of hospitalization in the experimental group. (Page 7, Line 150)

- Patients and therapist place the meaningful tasks in the meaningless time which selected in step 2. Also, patients and therapist create a timetable. Steps 1 to 3 were performed on the first day of intervention. The next day, the last step is to practice and occupational therapist intervention. (Page 8, Line 157-162)

6. Describe how the medical checkup criteria of the patient were checked and confirmed before undertaking the study.

**Answer> As your recommendation, we described the medical checkup criteria of the patient were checked before the study.**

- The ward nurse routinely conducts medical checkups such as chest x-ray, blood pressure, blood test, oxygen saturation, and BT for all inpatients regardless of research. This is to determine the severity of pneumonia and whether it is hemodynamically stable. Only patients who agreed to the study had their medical state reviewed by the medical staff of this study.

(Page 5-6, Line 106-110)

7. Describe all the tools used in the study with the rationality of choosing them in measuring the outcome in the method part shortly.

**Answer>** As your recommendation, we added explanation of secondary outcome measures shortly.

- Patient Health Questionnaire-9 (PHQ-9) …… A higher score indicates a better QOL [22].

(Page 9-10, Line 195-234)

8. Sample size calculation and the inclusion criteria would be good to cover in the same subheading. Please cite the paper referenced to calculate the sample for this study.

**Answer>** As your recommendation, we transferred sentence about sample size calculation from section for Statistical analysis to same section with inclusion criteria. Also, we cited the reference previous study about sample size.

- The sample size was….. LBI using G*Power 3.1.9. (Page 6, Line 127-132)

9. Also, add the limitation of a non-blinded trial in the limitation part.

**Answer>** As your recommendation, we added limitation about lacking blinding. Non-blinded trial could cause performance and detection bias. We assigned groups separately and use same outcome measurement and methods to reduce bias. In addition, data collection was conducted by two well-trained clinicians with more than 5 years of experience.

- Fourth, we considered a single blinded experiment, but it was not feasible due to manpower shortages and the environment of the isolation ward. This study is non-blinded trial could cause performance bias and detection bias. However, we did the following efforts to reduce the bias. We assigned patients to separate rooms to prevent group mixing, and same outcome measurement and methods were used for both groups. Also, two well-trained clinicians conducted data collection to reduce bias. (Page 17, Line 365-370)

10. The method and procedure of data collection and the questionnaire format need to be described in little detail in the method section.

**Answer>** As your comment, we added that contents to the method. Evaluations were conducted after admission and before discharge for both groups. All outcome measures were written in a google form, and patients evaluated with google form of table PC. This is a method often used in the non-face-to-face era. We attach some of the google forms that we used to help understand. Yellow section is outcome measurements of google form.

- Evaluations were conducted after admission and before discharge for both groups. All outcome measures were written in a google form, and patients evaluated with google form of table PC. (Page 11, Line 237-239)

**Reviewer #3:**

The authors evaluated a new protocol for time-use among patients suffering from infectious diesases requiring isolation which is very important in this current situation when the world is fighting for prevention and control of the ongoing COVID-19 pandemic. If enough evidence is generated through the intervention trials for improving mental health, occupational balance and quality of life during such crisis where patients require to stay isolated for long period of time from family and friend, it can be impactful for improving patient’s compliance to treatment even during convalescent period.

**Answer>** I appreciate for your kind review.

The manuscript may be considered for publication after addressing the following comments.

**Methods:**

Line 79: What assumptions were made for including 50 patients for this trial? Justification for this sample size need to be addressed. To study effectiveness of a new intervention protocol, a considerable number of samples needs to be included to provide a significant result of the analysis.

**Answer>** Previous studies were referred to for setting the sample size. Citations of previous studies referenced have been added. In addition, the sample size was justified through post analysis.

- The sample size was….. LBI using G*Power 3.1.9. (Page 6, Line 127-132)

Line 80: it is not clear if the patients were isolated in single cabin or ward. How were the study participants followed-up?

**Answer>** As your comments, we added information about ward types. All patients were isolated in ward for 4 patients. The same researcher followed up during hospitalization. We couldn't follow up after discharge.

- The two groups were assigned to wards separately so that they did not mix. (Page 5, Line 103)

Line 83: What measures taken to avoid bias? If a patient assigned to control group gets into contact with intervention group, there is a risk that control group participants may adapt some of the tasks assigned to intervention group. Hence was any measure taken to avoid the introduction of this bias?

**Answer>** This study was randomization control trial, but we could not be performed double blinded. So, it may cause bias. However, we tried to avoid bias through the following measures. Wards consisted of a hospital room used by patients who were not registered in the study and the experimental group, patients who were not registered in the study and the control group. The hospital that conducted the study operated 20 four-person rooms. As your comments, we added sentence as follow.

- The two groups were assigned to wards separately so that they did not mix. (Page 5, Line 103)

Line 97: How were the participants instructed or briefed about the intervention protocol. Was there any formal training provided to the participants in the intervention arm on the procedures to comply the intervention protocol?

**Answer>**

**Time use intervention :** The time-use intervention flow is explained through data similar with Figure 2 before starting. Occupational therapist and patients made timetable together. We attached intervention flow and timetable that we actually used for better understanding. Fill in the gray areas with the occupations selected in Step 2. Participants look at the timetable and implement the planned occupations. The therapist confirmed daily occupation performance.

**Education for self-activity** : We used the ‘Mind Care Guide for Persons with Infectious Diseases’ and the ‘Mental Health Guide for Body and Mind Recovery’ provided by the National Trauma Center in Korea as educational materials. Education includes the importance to maintain a regular life pattern along with stress relief methods and stabilization techniques. The picture is part of the educational material. It can be found on the National Trauma Center website (<https://www.nct.go.kr/>).

|  |  |
| --- | --- |

- Educational materials were used the ‘Mind Care Guide for Persons with Infectious Diseases’ and the ‘Mental Health Guide for Body and Mind Recovery’ provided by the National Trauma Center in Korea. It can be found on the National Trauma Center website (<https://www.nct.go.kr/>). Education includes the importance to maintain a regular life pattern along with stress relief methods and stabilization techniques. (Page 8, Line 169-172)

Line 101: How is the occupation selection done? Please define the steps to clarify.

**Answer>** Occupation selection is based on the results of K-LBI which our primary outcome measurement. There are 53 occupations in K-LBI. Activities that the subject is doing less than the desired time and activities that the subject is interested in are added. We attach a part of the English version of LBI for better understanding.

- Occupation selection is based on the results of K-LBI. There are 53 occupations in K-LBI. Activities that the subject is doing less than the desired time and activities that the subject is interested in are added. (Page 7, Line 153-156)

**Results**

Line 207: Delete “was” as it is abundant in this sentence.

**Answer>** As your recommendation, we deleted “was” in line 207.

- The change in the control group for all the occupational balance items not statistically significant (*p*=.05). (Page 13, Line 289)

Table 2- The statistical tests used in this analysis should be mentioned in the footnote with the appropriate level of significance.

**Answer>** As your recommendation, we added mention in the table 2, footnote, and figure3 with the appropriate level of significance. We set the level of significance to *<0.05, **<0.001. (Page 23-24, Figure3)

**Discussion:**

Line 229: Authors can emphasize on the scoring range found from previous studies that correlates with the level of stress.

**Answer>** ‘Correlates’ has been modified to ‘similar’. I am sorry to there was an error as English is not the main language of us. And I added the contents of the previous study.

- This finding similar with those of previous studies showing that time-use intervention is effective for persons with serious mental illness. Time-use intervention group increased their occupational balance by spending more per day in activity than the control group (p = .05) [11]. (Page 15, Line 323-326)

Line 261-266: Elaborate the discussion on how the QOL scores differ in different settings of isolation. For example, isolating in a single cabin, ward, or institute with having or not having activities involved during the isolation period.

**Answer>** The setting of the patients was the same as ward. We think your comment is very interesting. Unfortunately, we could not identify each patient's having or not having activities due to the lack of manpower and isolation environment. However, since the K-LBI score and QOL score of the experimental group are higher than the control group, it can be indirectly interpreted that having activities as much as desired is helpful for QOL.

**Conclusion:**

Line 291-294: Authors should emphasize the prospective impact of this new developed protocol of the time-use intervention as well as way forward in the field of mental health and challenges.

**Answer>** As your recommendation, we revised conclusion section.

- Isolated environment has had a negative impact on the occupational balance, mental health, and quality of life of COVID-19 patients. In addition, mental health problems can be long-term.

- Therefore, time-use intervention is needed to provide from the hospitalization period in patients with infectious diseases. (Page 18, 387-388)
