# Supplementary material for: Effects of a time-use intervention in isolated patients with coronavirus disease 2019: A randomized controlled study
Source: PLoS One. 2023 Jun 23;18(6):e0287118. doi: 10.1371/journal.pone.0287118 (PMC10289446; doi:10.1371/journal.pone.0287118)
Supplement: Attachment — Submitted filename: Response to Reviewers.docx [file pone.0287118.s008.docx]

**PLOS ONE - REVISION**

**Journal Requirements:**

1. Please review your reference list to ensure that it is complete and correct.

**Answer)** Reference has been corrected. Two references (previous numbers: 11 and 28) were deleted as duplicates, and two references (previous numbers: 12 and 15) were deleted as retracted articles. Also, reference 19 (existing number: 21) written in Korean has been revised into English.

**Additional Editor Comments:**

1. Please note that you must upload a completed CONSORT checklist as a supporting information file. Blank copies of these documents and information regarding CONSORT can be found via the following link: <http://www.consort-statement.org/>.

**Answer)** We uploaded the CONSORT checklist to the supporting information file.

2. Please upload a copy of the original trial study protocol as a supporting information file.

**Answer)** We uploaded the original trial study protocol to the supporting information file.

3. We suggest you thoroughly copyedit your manuscript for language usage, spelling, and grammar.

**Answer)** We reviewed and revised language usage, spelling, and grammar once again.
